# Betaine restores OPA1-mediated mitochondrial fusion in alveolar epithelial type 2 cells and ameliorates hyperoxic lung injury in newborn mice

**DOI:** 10.3389/fcell.2026.1937629

**Published:** 2026-09-29

**Authors:** Jufeng Zhang, Hongyu Xie, Hui Xu, Meiling Zhang, You You, Jingwen Lv, Li Wang, Hongping Xia

**Affiliations:** Department of Neonatology, Xinhua Hospital Affiliated to Shanghai Jiao Tong University School of Medicine, Shanghai, China

**Keywords:** alveolar type II epithelial cells, betaine, bronchopulmonary dysplasia, hyperoxia, OPA1

## Abstract

**Introduction:**

Bronchopulmonary dysplasia (BPD) is a common chronic respiratory disease in preterm infants, characterized by impaired lung development. Mitochondrial dysfunction of alveolar type II epithelial (AT2) cells plays a critical role in the pathogenesis of BPD. Betaine is an endogenous methyl donor with anti-inflammatory and antioxidant properties. Our previous study demonstrated that plasma betaine levels were significantly decreased in preterm infants with BPD. The aim of this study was to investigate the effect of betaine on mitochondrial dysfunction of AT2 cells in BPD.

**Methods:**

Hyperoxia-induced BPD mouse models and hyperoxia-exposed MLE-12 cells were used. Proteomic analysis was performed on lung tissues from BPD and control mice. Mitochondrial structure and function were assessed by transmission electron microscopy, immunofluorescence, Western blotting, flow cytometry, and related assays. The OPA1 inhibitor MYLS22 was used to evaluate the role of OPA1 in the protective effect of betaine.

**Results:**

Hyperoxia exposure induced mitochondrial structural damage in AT2 cells and impaired lung development in BPD mice. Proteomic analysis revealed that differentially expressed proteins were significantly enriched in mitochondrial dysfunction pathways in the lungs of BPD mice. Family with sequence similarity 162 member A (FAM162A), a mitochondria-associated protein involved in mitochondrial homeostasis, was prominently downregulated. Betaine significantly increased the expression of FAM162A and optic atrophy 1 (OPA1), suppressed the activating transcription factor 4 (ATF4) and C/EBP homologous protein (CHOP) mediated integrated stress response, and alleviated cell apoptosis in AT2 cells in BPD mice. In vitro, betaine increased the expression of FAM162A and OPA1, improved mitochondrial dysfunction and decreased cell apoptosis, and the effect was abolished by the OPA1 inhibitor MYLS22 in MLE-12 cells exposed to hyperoxia.

**Discussion:**

Our findings revealed that betaine ameliorated OPA1-mediated mitochondrial fusion in AT2 cells and alleviated hyperoxic lung injury in newborn mice, providing new insight for the prevention and treatment of BPD.

## Graphical Abstract

**Figure:**
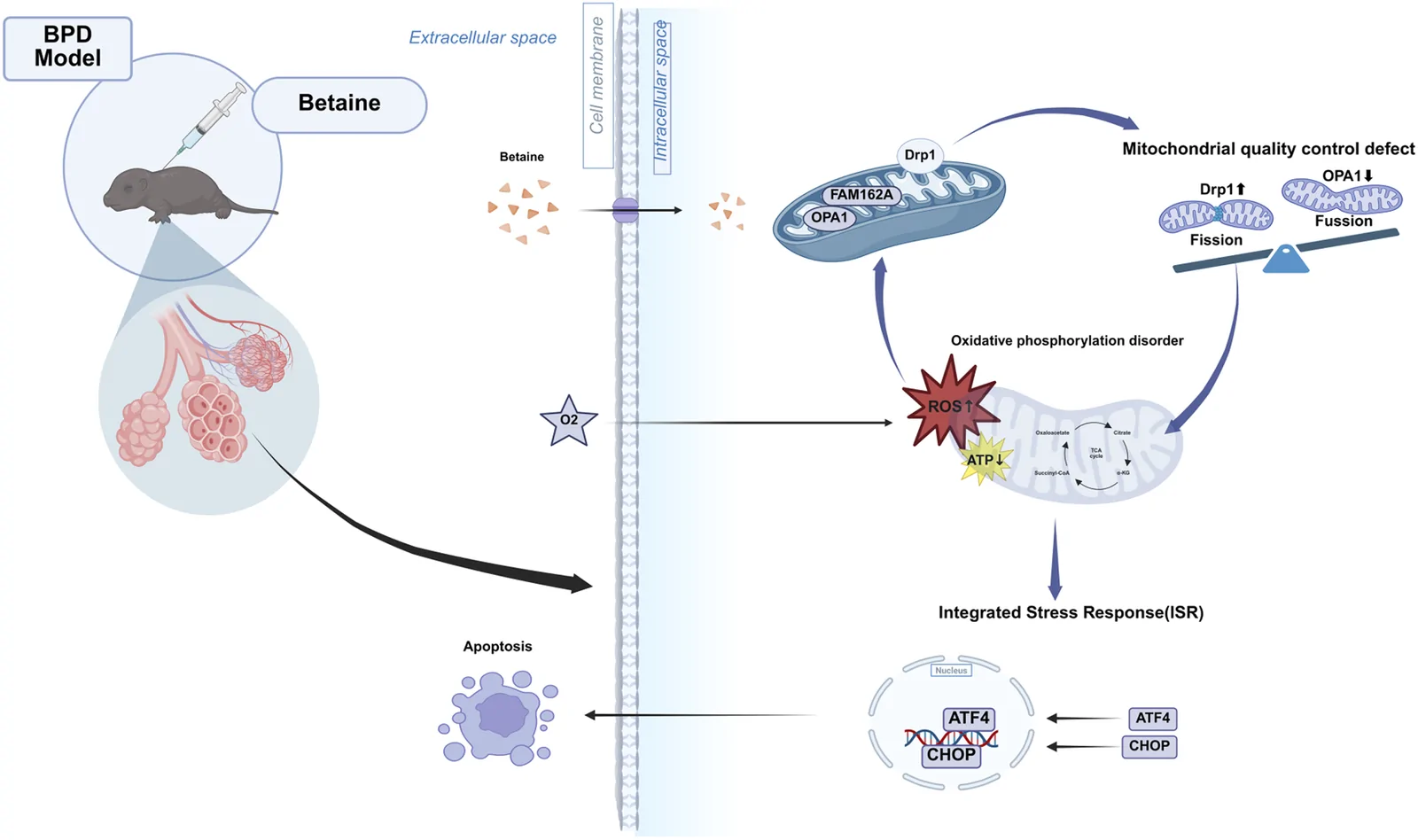
Betaine restores FAM162A and OPA1 expression, promotes OPA1-mediated mitochondrial fusion in AT2 cells, reduces oxidative stress, integrated stress response-related signaling and apoptosis, and ultimately ameliorates hyperoxia-induced lung injury in newborn mice. Created in BioRender. hongyu, X. (2026) https://BioRender.com/uv5y0w.

## 1 Introduction

Bronchopulmonary dysplasia (BPD) is a common chronic lung disease in preterm infants and is a major cause of long-term respiratory morbidity in preterm infants (Thebaud et al., 2019). BPD is characterized by impaired alveolarization and abnormal pulmonary vascular development, which are associated with placental dysfunction, hyperoxia, ventilator-induced lung injury, inadequate nutrition and abnormal pulmonary blood flow (Ambalavanan et al., 2026). Despite advances in neonatal intensive care, the incidence of BPD continues to rise (Bell et al., 2022). The management of BPD includes protective mechanical ventilation strategy, infection prevention, and nutritional support, etc. (Abiramalatha et al., 2022). However, the specific pharmacological interventions remain limited. Therefore, elucidating the molecular mechanisms underlying BPD is of great importance for identifying potential therapeutic targets and improving clinical outcome.

Alveolar type II (AT2) cells are essential for postnatal alveolar development and epithelial repair. In addition to their role in surfactant synthesis, AT2 cells serve as progenitor cells for alveolar epithelial regeneration and are highly dependent on mitochondrial metabolic activity to maintain cellular homeostasis (Wang et al., 2024). Mitochondrial dysfunction can disturb lung epithelial cell fate; mitochondrial complex I dysfunction activates the integrated stress response and impairs postnatal alveolar development (Han et al., 2023). Short-term hyperoxia impaired oxidative phosphorylation, reduced respiratory complex I and IV activity, and altered mitochondrial substrate utilization in cultured lung epithelial cells, and caused alveolar simplification in mice (Garcia et al., 2021). Mitochondrial dynamics, primarily including mitochondrial fusion and fission, are essential for maintaining mitochondrial morphology, bioenergetic function, and cellular homeostasis (Chen et al., 2023). Optic atrophy 1 (OPA1), a dynamin-related GTPase located in the inner mitochondrial membrane, regulates inner-membrane fusion and cristae organization. The balance between the long and short isoforms of OPA1 is particularly important for maintaining mitochondrial network integrity and respiratory function (Daumke and van der Laan, 2025). Disruption of OPA1 activity or processing can impair mitochondrial fusion, promote mitochondrial fragmentation and cristae disorganization, and increase cellular susceptibility to oxidative stress and apoptosis. Family with sequence similarity 162 member A (FAM162 A), also known as HGTD-P, is a stress-responsive mitochondrial protein originally identified as a downstream target of hypoxia-inducible factor-1α. Under hypoxic conditions, FAM162 A interacts with the voltage-dependent anion channel and promotes mitochondrial permeability transition, cytochrome c release, and caspase-dependent apoptosis, indicating that it directly transmits cellular stress signals to the mitochondrial apoptotic machinery (Lee et al., 2004). Subsequent evidence has suggested that the function of FAM162 A extends beyond the induction of apoptosis. FAM162 A has recently been localized predominantly to the mitochondrial inner membrane and cristae, where it contributes to the maintenance of cristae ultrastructure, mitochondrial bioenergetics, turnover, and stress resistance. Notably, FAM162 A deficiency results in a fragmented and less interconnected mitochondrial network, whereas its overexpression promotes a more elongated mitochondrial morphology. FAM162 A has also been reported to associate and interact with OPA1 and to regulate the balance between long and short OPA1 isoforms, thereby linking FAM162 A to OPA1-dependent mitochondrial fusion and cristae maintenance (Matamoros et al., 2026). Mitochondrial dynamics are increasingly recognized as important determinants of alveolar epithelial cell function during lung development. Hyperoxic exposure activates DRP1-mediated mitochondrial fission and induces glycolytic metabolic reprogramming in AT2 cells, suggesting that excessive mitochondrial fragmentation and bioenergetic impairment contribute to arrested alveolar development in BPD (Sun et al., 2024). However, whether FAM162 A participates in hyperoxia-induced mitochondrial dysfunction in AT2 cells and whether its effects involve OPA1-dependent mitochondrial fusion remain unclear.

Betaine is a naturally occurring metabolite with anti-inflammatory and antioxidant properties (Geng et al., 2025; Luo et al., 2025). Growing evidence indicates that betaine exerts protective effects in multiple disease models by modulating mitochondrial function, oxidative stress, and cellular stress responses (Khodayar et al., 2018; Veskovic et al., 2019). Our previous study revealed that plasma betaine levels at 36 weeks postmenstrual age were significantly lower in preterm infants with BPD than in those without BPD (You et al., 2024). However, the role of betaine in the pathogenesis of BPD remains unknown. Therefore, this study aimed to investigate whether betaine attenuates hyperoxia-induced lung injury by improving mitochondrial homeostasis and reducing AT2 cell apoptosis in BPD.

## 2 Materials and methods

### 2.1 Animal care and study design

We used a traditional mouse model of BPD induced by hyperoxia. Timed-pregnant C57BL/6 J mice were obtained from the Shanghai Laboratory, Animal Center Chinese Academy of Sciences, Shanghai, China. All animal experiments followed the ARRIVE guidelines and received approval from the Institutional Animal Care and Use Committee at Xinhua Hospital, affiliated with Shanghai Jiao Tong University School of Medicine (Approval No. XHEC-F-2024-025). The animals were kept in a SPF environment at a temperature of 23 °C ± 1 °C, following a standard 12-h light and dark cycle. Within 12 h of birth, the newborn mice were randomly divided into four groups: room air group (RA), room air with betaine group (RA + Bet), hyperoxia group (85%O_2,_ HO), and hyperoxia with betaine group (85%O_2,_ HO + Bet). Hyperoxia exposure was conducted in a specialized airtight plexiglass chamber, with oxygen concentrations continuously calibrated via a Miniox II oxygen monitor. For the intervention groups, betaine (50 mg/kg; MCE, HY-B0710) was administered via daily subcutaneous injections from postnatal day 1 (P1) to day 14 (P14) according to the literature (Zhang et al., 2026). To decrease oxygen toxicity in nursing dams, mothers were alternated between hyperoxic and room air environments every 24 h until tissue was collection on P7 and P14.

### 2.2 Lung proteomic profiling and bioinformatic analysis

Lung tissues were lysed with RIPA to extract total protein. After centrifugation at 12,000 × g for 25 min at 4 °C, the protein concentration in the supernatant was assessed with a BCA assay (Beyotime, P0010). For protein purification, 100 μL of each lysate was mixed with chilled methanol and centrifuged at 12,000 × g for 15 min. After collection, the protein pellets were dried in a vacuum centrifugal concentrator and then analyzed using liquid chromatography–tandem mass spectrometry (LC-MS/MS). Differentially expressed proteins (DEPs) were identified based on the predefined fold-change and statistical significance thresholds, and hierarchical clustering was used to visualize their expression patterns. Functional characterization of these DEPs was performed using the Gene Ontology (GO) database, focusing on biological processes, molecular functions, and cellular components. Emphasis was placed on identifying DEPs associated with mitochondrial fusion dynamics to elucidate their role in hyperoxia-induced lung injury.

### 2.3 Measurement of betaine levels in lung tissues

Betaine levels in lung tissues were measured by targeted liquid chromatography-tandem mass spectrometry (LC-MS/MS). Four mice were randomly selected from each of the RA and HO groups. Approximately 10 mg of right lung tissue was collected from each mouse and used for betaine detection. Lung tissue samples were weighed and mixed with 200 μL extraction solution. The samples were homogenized using a high-speed tissue grinder at 60 Hz for 60 s, and the homogenization procedure was repeated three times. The homogenates were then centrifuged at 10,000 rpm for 15 min at 4 °C. The supernatants were collected and transferred into autosampler vials for LC-MS/MS analysis. Chromatographic separation was performed on an Agilent 1,290 Infinity UHPLC system using an XBridge BEH Amide column. The mobile phase consisted of 10 mM ammonium formate (pH 3.5) and acetonitrile. The gradient elution program was as follows: 0–1 min, 98% B; 1–6 min, 98%–90% B; 6–7 min, 90% B; 7–10 min, 90%–85% B; 10–12 min, 85%–70% B; and 12–13 min, 70%–60% B, followed by a post-run time of 5 min. The flow rate was 0.35 mL/min, the injection volume was 2 μL, and the column temperature was maintained at 30 °C. Mass spectrometric detection was performed using an Agilent 6,470 Triple Quadruple MS/MS system in positive ion mode. For quantification, authentic betaine standard was used to prepare a stock solution at 1 mg/mL in water and stored at −20 °C. Working standard solutions were prepared by serial dilution with 80% methanol. Calibration curves were generated by plotting the standard concentration against the ratio of the betaine peak area to the internal standard peak area. Betaine concentrations in lung tissue samples were calculated from the corresponding calibration curve and normalized to tissue weight.

### 2.4 Pulmonary morphometric analysis

Following ketamine-induced anesthesia, neonatal mice were sacrificed at P7 or P14. Samples from the right lung were collected and preserved at −80 °C for protein measurement. To preserve lung architecture, the left lungs were subjected to pressure-controlled inflation (25 cmH_2_O) with 4% PFA for 15 min before being fixed overnight and paraffin-embedded. The lower lobe was sectioned transversely into 5 μm slices and stained with hematoxylin and eosin (H&E). Alveolar development was assessed using radial alveolar count (RAC) and the number of secondary septa. Lung sections stained with hematoxylin and eosin were observed under a light microscope, and representative lung parenchymal regions were analyzed while avoiding large airways and vessels. For each sample, three non-adjacent sections (separated by at least 100 μm to avoid double-counting the same structure) were selected for analysis. All images were captured using a light microscope (Olympus B201s) equipped with a digital camera. RAC was measured as described below: a perpendicular line was drawn from the center of a terminal bronchiole to the nearest pleura or interlobular septum. The number of alveoli intersected by this line was counted. For each section, five randomly selected terminal bronchioles were analyzed, yielding a total of 15 measurements per sample. Secondary septa were quantified at ×200 magnification. For each section, five randomly selected non-overlapping fields were examined. The number of secondary septa (defined as protrusions arising from the primary septa) was counted per field. A total of 15 fields per sample were analyzed. All quantitative analyses were performed in a blinded fashion. Data are expressed as mean ± SEM, with n = 4–6 samples per group at each time point.

### 2.5 Transmission electron microscopy (TEM)

At P14, 1 mm^3^ fresh lung tissue was fixed in 4% paraformaldehyde overnight at 4 °C, dehydrated in graded ethanol solutions at 4 °C, and embedded in acetone for 4 h at room temperature. Ultrathin sections (50–60 nm thickness) were prepared and double-stained with uranyl acetate and lead citrate for 30 min at room temperature. Lung tissue sections were examined using a transmission electron microscope (HITACHI, Tokyo, Japan) at 80 kV. AT2 cells were identified by the presence of characteristic lamellar bodies. Three animals per group were randomly selected for TEM analysis. For each animal, six randomly selected non-overlapping fields containing AT2 cells were captured at ×3,000 magnification for cell identification and counting, yielding a total of at least 6 AT2 cells analyzed per animal. At 100,00× magnification, the same AT2 cells were further examined for mitochondrial ultrastructural features, including cristae organization, matrix density, and mitochondrial swelling or vacuolation. Images were quantified using ImageJ. Mitochondrial size was assessed by manually tracing the outline of each mitochondrion to calculate its cross-sectional area (μm^2^), with a minimum of 15 mitochondria measured per animal. The mitochondrial fragmentation index (MFI) was determined by defining fragmented mitochondria as isolated, round-shaped mitochondria with a cross-sectional area of <1 μm^3^, and MFI was calculated as the percentage of fragmented mitochondria relative to the total mitochondria counted. All morphometric analyses were performed in a single-blinded manner, with the observer masked to group assignment by coded sample identifiers. Data are expressed as mean ± SEM.

### 2.6 Immunofluorescence staining

Lung sections embedded in paraffin were treated with primary antibodies targeting SP-C (Proteintech, 10774-1-AP, 1:200), ATF4 (CST, 11,815, 1:200), CHOP (Proteintech, 15204-1-AP, 1:200), OPA1 (Proteintech, 27733-1-AP, 1:200), and FAM162 A (Affinity, DF15168, 1:200) at 4 °C overnight. After washing, the sections were treated with the corresponding Coralite488-conjugated secondary antibodies, including anti-rabbit IgG (Proteintech, SA00013-2, 1:10,000) or anti-mouse IgG (Proteintech, SA00013-3, 1:10,000), for 1 h at room temperature. The nuclei were counterstained with DAPI (Servicebio, G1012, 1 μg/mL). Fluorescence images were obtained at ×200 magnification using a Pannoramic MIDI scanner, and double-positive cells were manually counted for quantitative analysis.

### 2.7 Western blotting

Lung tissues were lysed and sonicated using RIPA lysis buffer (Beyotime, P0013 K) supplemented with phenylmethylsulfonyl fluoride (PMSF, Beyotime, P1005-1). MLE-12 cells were gathered and lysed in the same buffer on ice for half an hour. After centrifuging at 12,000 × g for 25 min at 4 °C, the supernatants were taken for subsequent analysis. Protein levels were assessed with a BCA Protein Assay Kit (Beyotime, P0010). Equal protein quantities were resolved by 10% or 12.5% SDS-PAGE and subsequently transferred onto PVDF membranes (0.45 μm; Millipore Corporation, Billerica, MA, USA). After a 30-min room temperature block with block solution (Beyotime, P0252), the membranes were exposed to primary antibodies against Bax (CST, 2,772, 1:2000), Bcl-2 (CST, 3,498, 1:2000), Cleaved caspase-3 (CST, 9,664, 1:2000), ATF4 (CST, 11,815, 1:1,000), CHOP (Proteintech, 15204-1-AP, 1:1,000), OPA1 (Proteintech, 27733-1-AP, 1:5,000), FAM162 A (Biorbyt, orb1880878, 1:1,000), p-DRP1 (Affinity, DF2980, 1:2000), DRP1 (Affinity, DF7037, 1:2000), and β-actin (Proteintech, 60008-1-AP, 1:10,000) overnight at 4 °C. The membranes were incubated with the appropriate secondary antibodies for 2 h at room temperature after been washed with Tris-buffered saline containing 0.1% Tween-20 (TBS-T). Protein bands were visualized using enhanced chemiluminescence reagents from Millipore Corporation (Billerica, MA, USA), and ImageJ software was used to quantify the band intensities (NIH, Bethesda, MD, USA).

### 2.8 Cell culture and culture conditions

The MLE-12 cell line was obtained from the Cell Bank of the Chinese Academy of Sciences (Shanghai, China). The cells were cultured in Minimum Essential Medium-α (Gibco, Grand Island, NY, USA) supplemented with 10% fetal bovine serum and 1% penicillin/streptomycin at 37 °C in a humidified atmosphere containing 5% CO_2_. Cells exposed to 21% oxygen served as the control group, whereas cells exposed to 85% oxygen were assigned to the hyperoxia (HO) group. Cells were planted and treated with betaine before been exposed to high oxygen levels for the experiments. After 48 h of exposure to 85% oxygen, cells were harvested for subsequent analyses. MYLS22 (TargetMol, T9127), a pharmacological inhibitor of OPA1 GTPase activity, was applied at a concentration of 50 μM. MLE-12 cells were randomly divided into six groups: normoxia group (21%O_2_, NO), normoxia with betaine group (21%O_2_, NO + Bet), hyperoxia group (85%O_2_, HO), hyperoxia with betaine group (85%O_2_, HO + Bet), hyperoxia with MYLS22 group (85%O_2_, HO + MYLS22), and hyperoxia with MYLS22 and betaine group (85%O_2_, HO + MYLS22 + Bet).

### 2.9 Cell counting kit-8 (CCK-8) assay

MLE-12 cells were placed in 96-well plates with a density of 5 × 10^3^ cells per well, and exposed to betaine at concentrations of 0, 5, 10, 20, 40, 80, and 160 mM for 48 h. Then, 10 μL of CCK-8 reagent (Beyotime, C0038) was added to each well, and the cells were incubated for an extra 2 h. Absorbance at 450 nm was measured with a Multiskan FC microplate reader (Thermo Fisher Scientific, Waltham, MA, USA).

### 2.10 Superoxide dismutase (SOD) and glutathione peroxidase (GSH-Px) measurement

The levels of SOD and GSH-Px were determined using commercial assay kits (Beyotime, S0101 S and S0056) according to the manufacturer’s instructions. Absorbance was measured with a Multiskan FC microplate reader (Thermo Fisher Scientific, Waltham, MA, USA).

### 2.11 Mitochondrial mass assessment

Mitochondrial mass was measured using MitoTracker Green (Beyotime, C1048). A stock solution of 1 mM was made by dissolving MitoTracker Green in dry DMSO and further diluted in pre-warmed culture medium to achieve the final working concentration of 100 nM. Following the removal of the culture medium, MLE-12 cells were incubated with the working solution at 37 °C for 30 min. The staining solution was then replaced with fresh pre-warmed culture medium, and the cells were instantly examined under a fluorescence microscope at the correct excitation/emission wavelengths for green fluorescence (Ex 490 nm/Em 516 nm). The mean fluorescence intensity of each image was quantified using ImageJ software under the same measurement settings. For each assay, the measured fluorescence intensity was normalized to the mean value of the normoxia (NO) group, which was set to 1, and the results were expressed as relative fluorescence intensity.

### 2.12 Mitochondrial membrane potential assay

Mitochondrial membrane potential (MMP, ΔΨm) was evaluated with the JC-1 Enhanced Mitochondrial Membrane Potential Assay Kit (Beyotime, C2003S). MLE-12 cells were collected and resuspended in 0.5 mL of culture medium, then an equal volume of JC-1 staining working solution was subsequently added. After been gently mixed, the cells were incubated at 37 °C for 20 min. The stained cells were then centrifuged at 600 *g* for 4 min at 4 °C, washed twice with JC-1 staining buffer, and resuspended in a suitable buffer volume for flow cytometric analysis. Low ΔΨm was indicated by green fluorescence from JC-1 monomers, while high ΔΨm was indicated by red fluorescence from JC-1 aggregates. Mitochondrial membrane potential was assessed using the ratio of red to green fluorescence intensity.

### 2.13 Detection of total intracellular ROS levels in MLE-12 cells

Intracellular ROS levels in total were assessed with a Reactive Oxygen Species Assay Kit (Beyotime, S0033). After treatment, MLE-12 cells were washed with a serum-free medium and incubated with a 10 µM DCFH-DA working solution (1:1,000 dilution in serum-free medium) at 37 °C for 30 min in darkness. Once incubation was complete, the cells were washed thrice with a serum-free medium to get rid of surplus probe. Fluorescence images were acquired using an inverted fluorescence microscope, with ROS fluorescence detected at excitation/emission wavelengths of 488/525 nm. Image analysis was performed using ImageJ software, and fluorescence intensity was corrected for background before statistical analysis. The measured fluorescence intensity was normalized to the mean value of the normoxia (NO) group, which was set to 1, and the results were expressed as relative fluorescence intensity.

### 2.14 Detection of intracellular ATP levels in MLE-12 cells

Intracellular ATP levels in MLE-12 cells were assessed with an ATP Assay Kit (Beyotime, S0026). Each group of MLE-12 cells was seeded at an identical density. The culture medium was discarded after treatment, and the cells were lysed on ice with 200 µL of prechilled ATP assay lysis buffer per well. The lysates were centrifuged at 12,000 × g for 5 min at 4 °C, and the supernatants were collected for analysis. A standard curve for ATP was created by diluting a 0.5 mM ATP standard with lysis buffer to achieve final concentrations between 0.01 and 10 µM. The ATP working reagent was created by mixing the assay reagent and dilution buffer in a 1:9 proportion. For the measurement, 100 µL of this working reagent was added to each well of a 96-well white plate. Following a 3–5 min incubation at room temperature, 20 µL of either sample or standard was promptly added and mixed. Chemiluminescence was detected using a luminometer, and ATP concentrations were calculated based on the standard curve.

### 2.15 Flow cytometric analysis of apoptosis

MLE-12 cells were harvested after the indicated treatments. Cells were collected using trypsin without EDTA and washed twice with ice-cold phosphate-buffered saline (PBS). After centrifugation, the cell pellets were resuspended in binding buffer and stained with Annexin V-FITC and propidium iodide (PI) using an Annexin V-FITC/PI Apoptosis Detection Kit (Yeasen, 40302ES60) according to the manufacturer’s instructions. Briefly, cells were incubated with Annexin V-FITC and PI for 15 min at room temperature in the dark and immediately analyzed using a Beckman Coulter flow cytometer. The percentage of apoptotic cells was calculated by combining the early apoptotic (Annexin V^+^/PI^−^) and late apoptotic (Annexin V^+^/PI^+^) populations.

### 2.16 RNA extraction and quantitative reverse-transcription PCR (RT–qPCR)

MLE-12 cells were seeded in six-well plates and cultured under different experimental conditions. After treatment, cells were washed twice with ice-cold phosphate-buffered saline (PBS). Total RNA was extracted using the SteadyPure Rapid RNA Extraction Kit (Accurate Biology, AG21023) according to the manufacturer’s protocol. RNA concentration and purity were measured using a NanoDrop spectrophotometer, and samples with an A260/A280 ratio of 1.8–2.0 were used for reverse transcription. Equal amounts of total RNA were reverse-transcribed into cDNA using the Hifair® AdvanceFast first Strand cDNA Synthesis SuperMix for qPCR (Yeasen, 11156 ES) following the manufacturer’s instructions. Quantitative real-time PCR was performed using the Hieff® qPCR SYBR Green Master Mix (Yeasen, 11202 ES) on a real-time PCR detection system. Each sample was analyzed in technical triplicate. The amplification conditions were as follows: an initial denaturation step at 95 °C for 30 s, followed by 40 cycles of denaturation at 95 °C for 10 s and annealing/extension at 60 °C for 30 s. A melting curve analysis was subsequently performed to evaluate the specificity of amplification.

The primer sequences were listed below:

Mouse Fam162a.

Forward: 5′-AAG​AAC​AAG​CTC​CGG​GTG​AA-3′

Reverse: 5′-CAT​GTC​TTT​TCG​CAG​CCT​TCT-3′

Mouse Actb.

Forward: 5′-CAT​CCG​TAA​AGA​CCT​CTA​TGC​CAA​C-3′

Reverse: 5′-ATG​GAG​CCA​CCG​ATC​CAC​A-3′

Actb was used as the internal reference gene. Relative Fam162a mRNA expression levels were calculated using the 2^−ΔΔCt^ method.

### 2.17 Statistical analysis

Image acquisition and quantitative analyses, including immunofluorescence cell counting, TEM mitochondrial scoring, RAC and secondary septa counting, and Western blot densitometry, were performed by investigators blinded to group assignment. The experimental unit and exact sample size (*n*) for each analysis are specified in the corresponding figure legends. For cell-based experiments, *n* represents the number of independent biological experiments rather than the number of images or technical replicates. Measurements obtained from multiple images or fields within the same biological sample were averaged to generate a single value for statistical analysis.

Statistical analyses were performed using GraphPad Prism 9.0. The normality of the data was assessed using the Shapiro–Wilk test, and homogeneity of variance was evaluated using the Brown–Forsythe test. For two-factor analyses, the normality of the model residuals was assessed. Data satisfying the assumptions of normality and homogeneity of variance were analyzed using one-way or two-way analysis of variance (ANOVA), as appropriate, followed by Tukey’s multiple-comparisons test. For single-factor comparisons involving three or more groups, data that did not satisfy the normality assumption were analyzed using the Kruskal–Wallis test followed by Dunn’s multiple-comparisons test. Normally distributed data are presented as the mean ± standard deviation (SD), whereas non-normally distributed data are presented as the median with interquartile range. All tests were two-sided, and a *p* value <0.05 was considered statistically significant (*p < 0.05, **p < 0.01).

## 3 Results

### 3.1 Hyperoxia induces mitochondrial damage and impairs lung development in newborn mice

In a classical hyperoxia-induced BPD mouse model (Figure 1A), H&E staining showed that hyperoxia-exposed mice exhibited typical pathological features of BPD at both P7 and P14, including alveolar simplification, reduced radial alveolar counts (RAC) and secondary septa (Figures 1B–D). TEM revealed that hyperoxia induced ultrastructural abnormalities, including mitochondrial swelling and vacuolation, nuclear pyknosis, loss of lamellar corpuscles, and disruption of cristae structure with morphological transition from fusiform to spherical in AT2 cells of BPD mice (Figure 1E). Hyperoxia significantly reduced mitochondrial size and aspect ratio, while markedly increasing the mitochondrial fragmentation index, indicating a shift toward smaller, rounder, and more fragmented mitochondria (Figures 1F–H). Endogenous betaine levels were significantly reduced in the lung tissue of BPD mice (Figure 1I). These results indicate that hyperoxia induces mitochondrial structural impairment in AT2 cells and impaired lung development in newborn mice.

**FIGURE 1:**
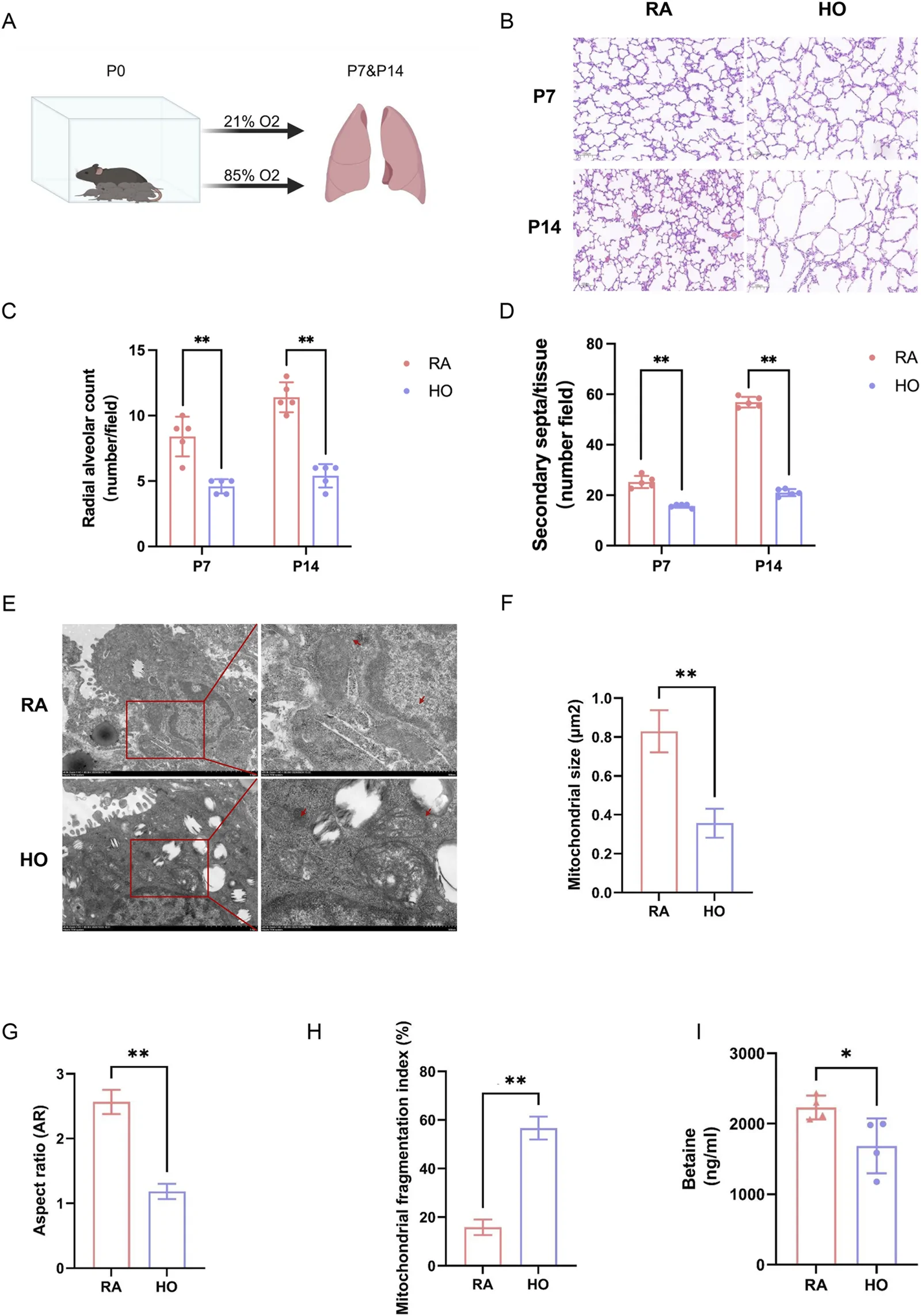
Hyperoxia induces mitochondrial damage and impairs lung development in newborn mice. **(A)** Schematic diagram of the hyperoxia-induced BPD mouse model. Newborn mice were exposed to 85% oxygen from postnatal day 1 (P1) to P14, with room air-exposed littermates serving as controls. Created in BioRender. hongyu, X. (2026) https://BioRender.com/4zi51wd. **(B)** Representative hematoxylin and eosin (H&E)-stained lung sections from RA (room air) and HO (hyperoxia) mice at P7 and P14. Scale bar = 100 μm. RA was defined as room air and HO as hyperoxia. **(C)** Quantification of radial alveolar count (RAC) in RA and HO mice at P7 and P14. Data are presented as mean ± SD; n = 5 animals per group. **(D)** Quantification of secondary septa number in lung sections from RA and HO mice at P7 and P14. Data are presented as mean ± SD; n = 5 animals per group. **(E)** Representative transmission electron microscopy (TEM) images of AT2 cells identified by characteristic lamellar bodies in lung tissues from RA and HO mice at P14, showing mitochondrial ultrastructural morphology. Arrows indicate mitochondria. Scale bars: 2 μm in low-magnification images and 500 nm in high-magnification images. n = 3 animals per group. **(F)** Quantitative measurement of AT2 cells mitochondrial size in each group (n = 30/area). **(G)** Quantitative measurement of AT2 cells mitochondrial aspect ratio (AR) in each group (n = 30/area). **(H)** Quantification of the mitochondrial fragmentation index. Measurements were performed using transmission electron microscopy (TEM) images of AT2 cells. **(I)** Quantification of endogenous betaine levels in lung tissues from RA and HO mice, showing a significant decrease in the BPD group. Data are presented as mean ± SD (n = 4 animals per group). Data are presented as mean ± SD. *p < 0.05, **p < 0.01 for the indicated comparisons.

### 3.2 Proteomic analysis reveals mitochondrial dysfunction-related alterations in the lungs of BPD mice

To systematically identify the molecular targets underlying hyperoxia-induced mitochondrial dysfunction, we performed proteomic analysis on the lung tissues of BPD and control mice. A total of 1,458 proteins were commonly identified in both groups, among which 56 were differentially expressed proteins (DEPs, fold change >1.5, *p* < 0.05), including 40 downregulated and 16 upregulated proteins in the BPD group (Figures 2A,B). Subcellular localization analysis showed that these DEPs were distributed across multiple cellular compartments, including mitochondria, suggesting the involvement of mitochondria-associated alterations in hyperoxia-induced lung injury (Figure 2C). GO enrichment analysis further showed that these DEPs were mainly enriched in mitochondrial inner membrane complexes, respiratory chain function, and ATP synthase-related complexes, collectively suggesting extensive impairment of mitochondrial energy metabolism (Figure 2D). Notably, mitochondria-associated FAM162 A was downregulated in the lungs of BPD mice compared with control mice, with a BPD/control expression ratio of 0.539, corresponding to an approximately 1.85-fold decrease (Figure 2E; Supplementary Table S1). Among the 56 DEPs, FAM162 A ranked fourth according to the magnitude of fold change but ranked 44th according to nominal p-value (p = 0.040). Therefore, FAM162 A was not among the most statistically significant DEPs identified by proteomic analysis. FAM162 A was selected for further investigation because of its mitochondrial inner membrane and cristae localization and its reported involvement in mitochondrial structural maintenance and OPA1-associated mitochondrial dynamics. Protein interaction network analysis further highlighted the association of these DEPs with mitochondrial dysfunction. (Figure 2F). These findings suggest that FAM162 A may participate in hyperoxia-induced mitochondrial dysfunction, and its role remains to be explored.

**FIGURE 2:**
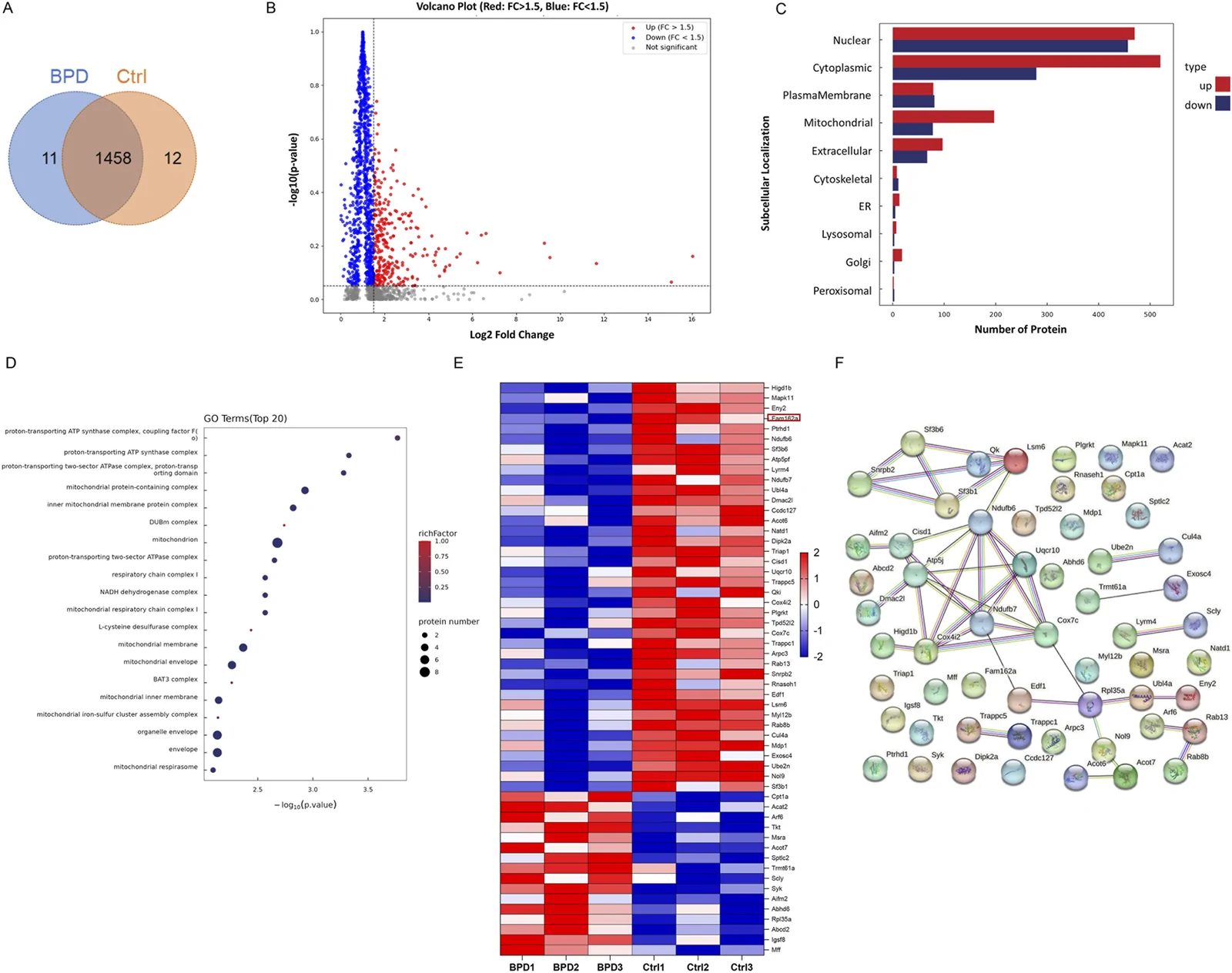
Proteomic analysis reveals mitochondrial dysfunction-related alterations in the lungs of BPD mice. **(A)** Venn diagram showing the overlap of identified proteins between the BPD and control groups. A total of 1,458 proteins were identified in common, with 11 unique to the BPD group and 12 unique to the control group. **(B)** Volcano plot of differentially expressed proteins between BPD and control groups. Proteins with fold change >1.5 and *p* < 0.05 are highlighted. A total of 56 differentially expressed proteins were identified, with 40 downregulated and 16 upregulated in the BPD group. **(C)** Subcellular localization map of identified proteins. **(D)** GO Cellular Component enrichment analysis of the top 20 enriched terms for differentially expressed proteins. **(E)** Heatmap displaying the differential expression of mitochondria-related proteins between the BPD and control groups. **(F)** Protein interaction network of differentially expressed mitochondrial proteins, constructed using the STRING database.

### 3.3 Betaine ameliorates mitochondrial structural abnormalities and attenuates hyperoxic lung injury in newborn mice

The betaine administration protocol is illustrated in Figure 3A. Hyperoxia significantly reduced body weight in BPD mice at both P7 and P14, whereas betaine treatment attenuated this reduction (Figure 3B). H&E staining showed that hyperoxia markedly reduced RAC and decreased the number of secondary septa in the lungs of BPD mice at both P7 and P14, indicating impaired alveolar development. Betaine ameliorated these histopathological changes, with higher RAC and more secondary septa number compared with the BPD group (Figures 3C–E). In addition, hyperoxia significantly decreased the levels of antioxidant enzymes, including SOD and GSH-Px, in the lungs of BPD mice, while betaine attenuated these reductions (Figures 3F,G). TME further showed that betaine ameliorated hyperoxia-induced ultrastructural abnormalities and improved mitochondrial morphology (Figure 3H). Betaine treatment significantly increased the mitochondrial aspect ratio and reduced the mitochondrial fragmentation index under hyperoxic conditions (Figures 3I–K). These findings indicate that betaine attenuates hyperoxia-induced alveolar simplification and promotes lung development in newborn mice.

**FIGURE 3:**
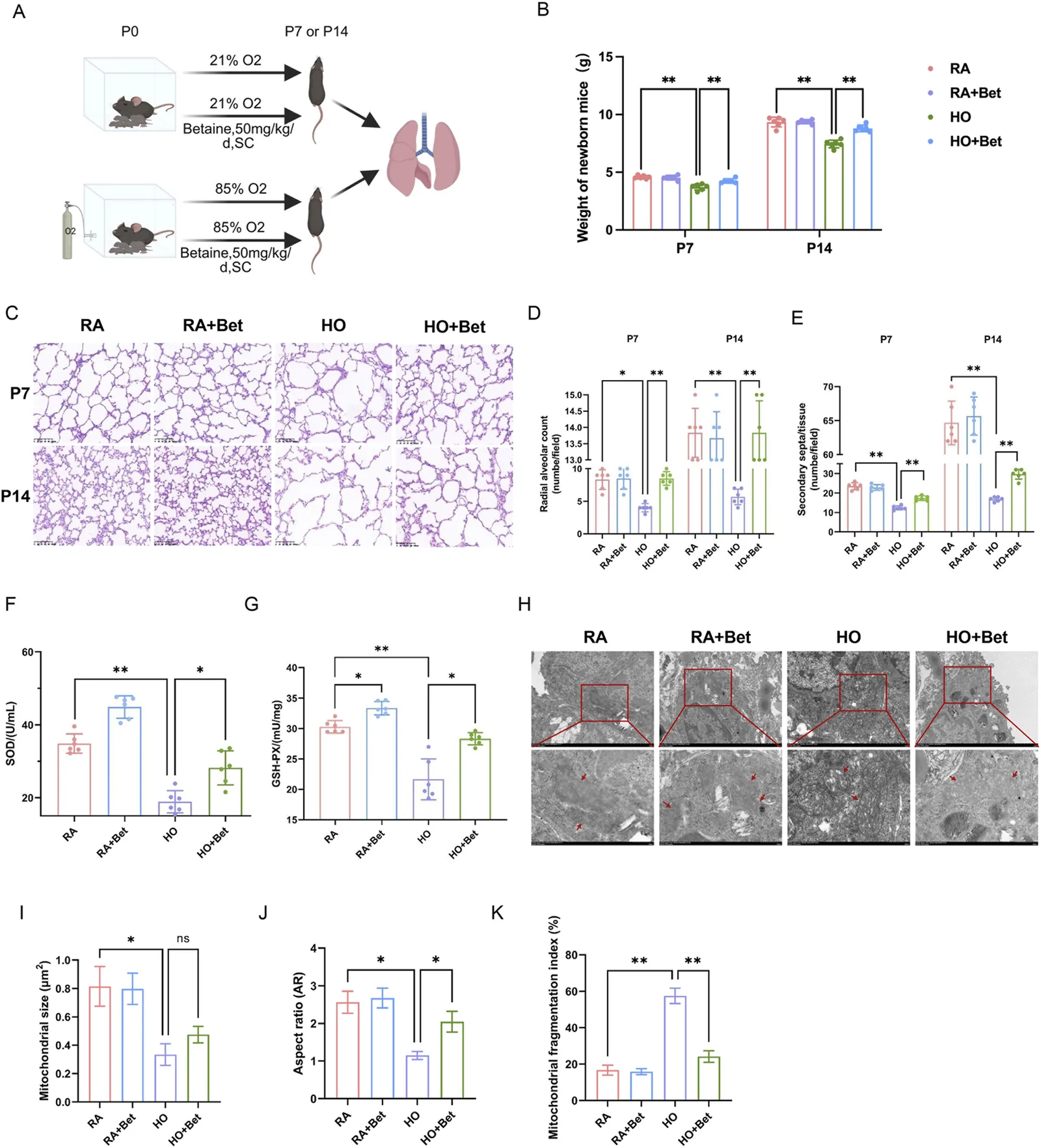
Betaine ameliorates mitochondrial structural abnormalities and attenuates hyperoxic lung injury in newborn mice. **(A)** Schematic diagram of the experimental design. Newborn mice were randomly divided into four groups: room air group (RA), room air with betaine group (RA + Bet), hyperoxia group (85%O2, HO), and hyperoxia with betaine group (HO + Bet). Betaine was administered subcutaneously at 50 mg/kg/day from P1 until tissue collection at P7 or P14. **(B)** Body weights of mice from the four groups at P7 and P14. Data are presented as mean ± SD; n = 6 per group. **(C)** Representative H&E-stained lung sections from each group at P7 and P14. Magnification: ×200. Scale bar = 100 μm. **(D,E)** Quantification of radial alveolar count (RAC) and secondary septa number in lung sections from each group. Data are presented as mean ± SD; n = 6 per group. **(F,G)** Quantification of superoxide dismutase (SOD) and glutathione peroxidase (GSH-Px) levels in lung tissues from each group, detected by microplate reader. Data are presented as mean ± SD; n = 6 per group. **(H)** Representative TEM images showing mitochondrial morphology in AT2 cells identified by characteristic lamellar bodies in lung tissues from each group at P14. Arrows indicate mitochondria. Scale bars: 2 μm in low-magnification images and 500 nm in high-magnification images. n = 3 animals per group. **(I)** Quantitative measurement of AT2 cells mitochondrial size in each group (n = 30/area). **(J)** Quantitative measurement of AT2 cells mitochondrial aspect ratio (AR) in each group (n = 30/area). **(K)** Quantification of the mitochondrial fragmentation index. Measurements were performed using transmission electron microscopy (TEM) images of AT2 cells. Data are presented as mean ± SD. *p < 0.05, **p < 0.01 for the indicated comparisons.

### 3.4 Betaine ameliorates mitochondrial dysfunction in hyperoxia-exposed MLE-12 cells

CCK-8 assay was performed to evaluate the effect of different concentrations of betaine on MLE-12 cell viability, and 10 mM betaine was selected for subsequent experiments (Figure 4A). Flow cytometric analysis of JC-1 staining showed that betaine attenuated the hyperoxia-induced loss of mitochondrial membrane potential in MLE-12 cells (Figures 4B,C). MitoTracker Green staining further demonstrated that betaine alleviated the hyperoxia-induced reduction in mitochondrial content (Figures 4D,E). In addition, betaine significantly reduced intracellular ROS accumulation and ameliorated ATP levels under hyperoxic conditions (Figures 4F–H). Collectively, these findings demonstrate that betaine exerts protective effects against hyperoxia-induced mitochondrial dysfunction in MLE-12 cells.

**FIGURE 4:**
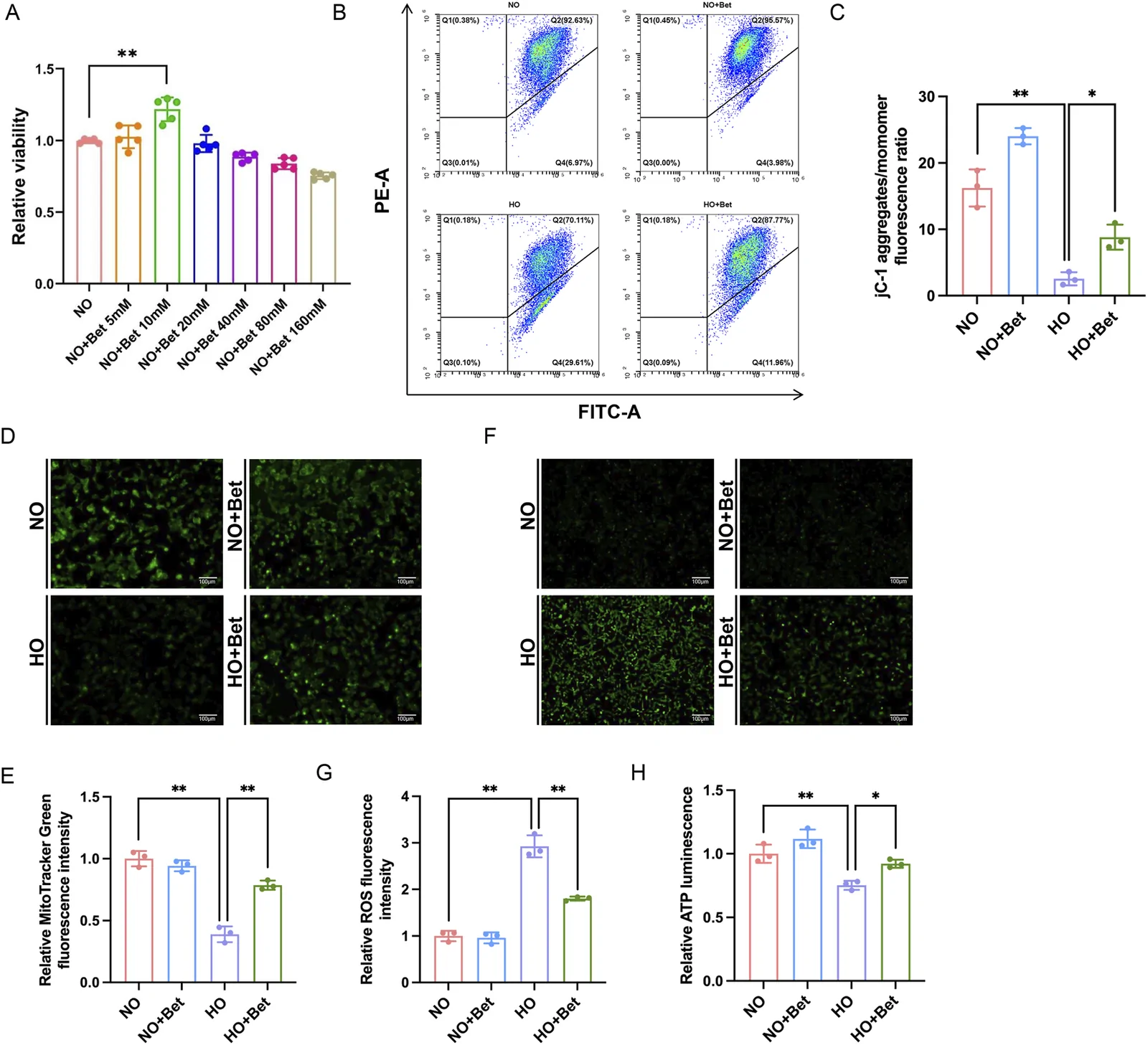
Betaine ameliorates mitochondrial dysfunction in hyperoxia-exposed MLE-12 cells. **(A)** Cell viability of MLE-12 cells treated with different concentrations of betaine (5–160 mM) for 48 h under normoxic conditions, assessed by CCK-8 assay. Data are presented as mean ± SD; n = 5 per group. For panels Created in BioRender. hongyu, X. (2026) https://BioRender.com/ylqzvdr.**(B–H)**, MLE-12 cells were divided into four groups: normoxia (21% O_2_, NO), normoxia + betaine (21% O_2_, NO + Bet), hyperoxia (85% O_2_, HO), and hyperoxia + betaine (85% O_2_, HO + Bet). **(B)** Representative flow cytometric analysis of mitochondrial membrane potential in MLE-12 cells using JC-1 staining. The upper-right population represents JC-1 aggregates with high mitochondrial membrane potential, whereas the lower-right population represents JC-1 monomers with reduced mitochondrial membrane potential. **(C)** Quantification of the JC-1 aggregates/monomers fluorescence ratio. Data are presented as mean ± SD; n = 3 per group. **(D)** Representative fluorescence images of mitochondrial mass detected by MitoTracker Green staining in MLE-12 cells from each group. Scale bar = 100 μm. **(E)** Quantification of relative MitoTracker Green fluorescence intensity normalized to the normoxia group. Data are presented as mean ± SD; n = 3 per group. **(F)** Representative fluorescence images of ROS levels detected by DCFH-DA staining in MLE-12 cells from each group. Scale bar = 100 μm. **(G)** Quantification of relative ROS fluorescence intensity normalized to the normoxia group. Data are presented as mean ± SD; n = 3 per group. **(H)** Quantification of relative ATP levels detected by luminescence assay, normalized to the normoxia group. Data are presented as mean ± SD; n = 3 per group. Data are presented as mean ± SD. *p < 0.05, **p < 0.01 for the indicated comparisons.

### 3.5 Betaine regulates the expression of mitochondrial dynamics-related proteins in the lung of BPD mice

Western blot analysis showed that hyperoxia decreased FAM162 A expression, whereas betaine treatment increased its expression in the lungs of BPD mice (Figures 5A,B). In addition, hyperoxia reduced the expression of the mitochondrial fusion-related protein OPA1 and increased the level of the mitochondrial fission-related protein p-Drp1. Betaine treatment increased OPA1 expression and reduced p-DRP1 levels (Figures 5C–E). Immunofluorescence analysis further revealed that betaine increased the expression of FAM162 A (Figures 5F,G) and OPA1 (Figures 5H,I) in the lungs of BPD mice. These findings suggest that betaine alleviates the imbalance in mitochondrial dynamic in the lungs of BPD mice, accompanied by increased FAM162 A and OPA1 expression and reduced p-DRP1 levels. To further determine whether the reduction of FAM162 A occurred at the transcriptional level, we examined FAM162 A mRNA expression by qPCR. Hyperoxia significantly decreased the mRNA level of FAM162 A in MLE-12 cells, whereas betaine treatment markedly restored FAM162 A mRNA expression under hyperoxic conditions. In contrast, betaine did not significantly alter FAM162 A mRNA expression under normoxic conditions (Supplementary Figure S1). These results suggest that hyperoxia-induced FAM162 A downregulation is at least partly associated with transcriptional suppression, and that betaine may restore FAM162 A expression at the mRNA level.

**FIGURE 5:**
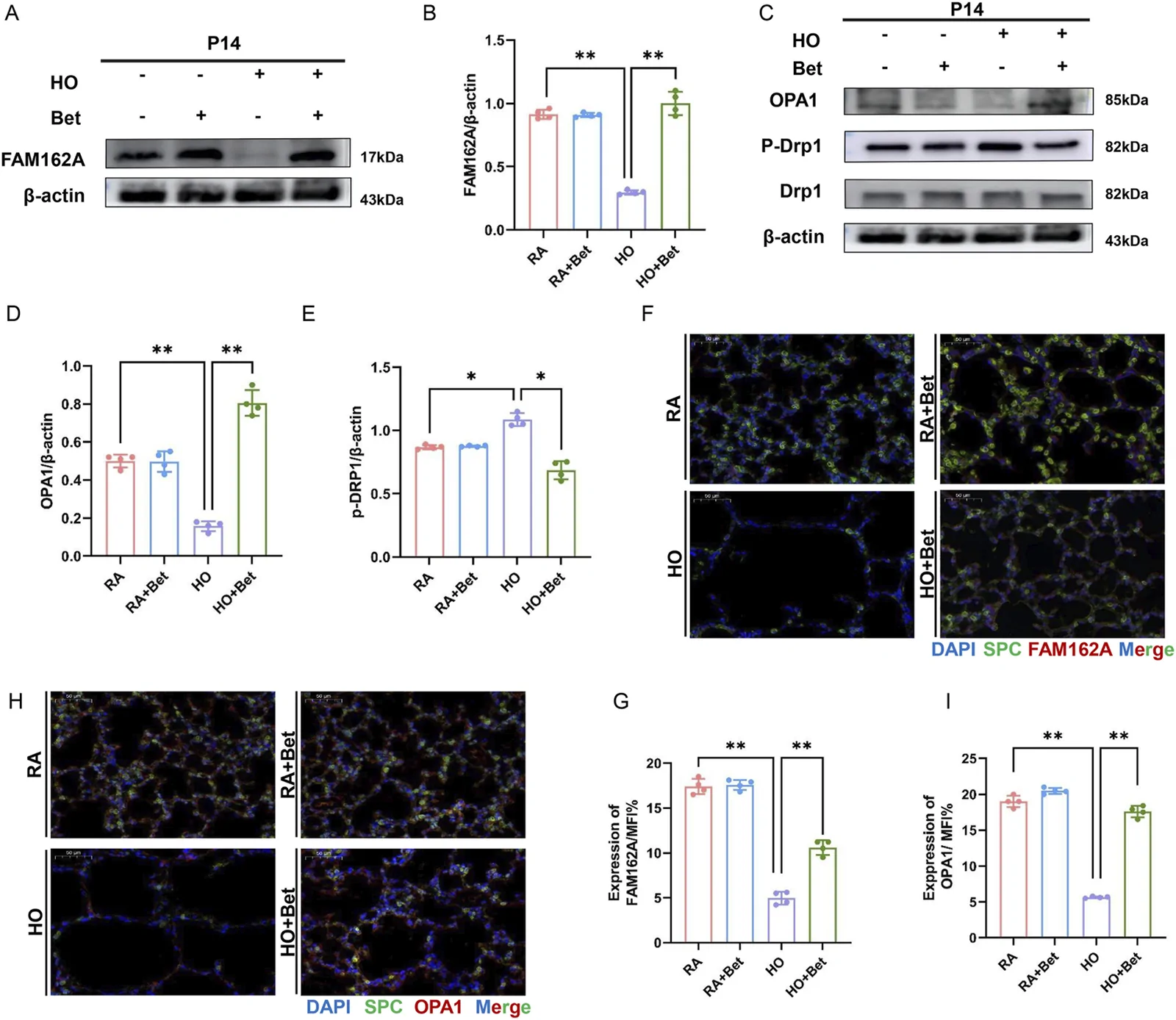
Betaine regulates the expression of mitochondrial dynamics-related proteins in the lung of BPD mice. **(A)** Representative Western blot images of FAM162 A protein expression in lung tissues from each group at P14. **(B)** Quantification of FAM162 A protein level normalized to β-actin by densitometric analysis. Data are presented as mean ± SD; n = 4 per group. **(C)** Representative Western blot images of OPA1, p-Drp1, and Drp1 protein expression in lung tissues from each group at P14. **(D,E)** Quantification of OPA1 and p-Drp1 protein levels normalized to β-actin by densitometric analysis. Data are presented as mean ± SD; n = 4 per group. **(F)** Representative immunofluorescence images showing FAM162 A localization in AT2 cells in lung tissues from each group at P14. Scale bar = 50 μm. **(G)** Quantification of FAM162 A-positive AT2 cells. Data are presented as mean ± SD; n = 4 per group. **(H)** Representative immunofluorescence images showing OPA1 localization in AT2 cells in lung tissues from each group at P14. Scale bar = 50 μm. **(I)** Quantification of OPA1-positive AT2 cells. Data are presented as mean ± SD; n = 4 per group. Data are presented as mean ± SD. *p < 0.05, **p < 0.01 for the indicated comparisons.

### 3.6 Betaine attenuates integrated stress response and apoptosis of AT2 cells in the lung of mice with BPD

Western blot analysis showed that hyperoxia increased the expression of ATF4 and the pro-apoptotic protein Bax, while decreasing the expression of the anti-apoptotic protein Bcl-2 in the lungs of mice with BPD. However, betaine treatment was associated with reduced ATF4 and Bax expression and increased Bcl-2 expression (Figures 6A–D). Immunofluorescence analysis further revealed that hyperoxia increased ATF4 and CHOP expression in SP-C-positive AT2 cells in the lungs of BPD mice, whereas betaine attenuated ATF4 and CHOP expression and increased SP-C expression (Figures 6E–H). Flow cytometric analysis further confirmed that betaine attenuated hyperoxia-induced apoptosis in MLE-12 cells, whereas MYLS22 treatment partially reversed this protective effect (Supplementary Figures S2A, B). These findings suggest that betaine alleviates hyperoxia-induced integrated stress response and apoptosis both *in vivo* and *in vitro*.

**FIGURE 6:**
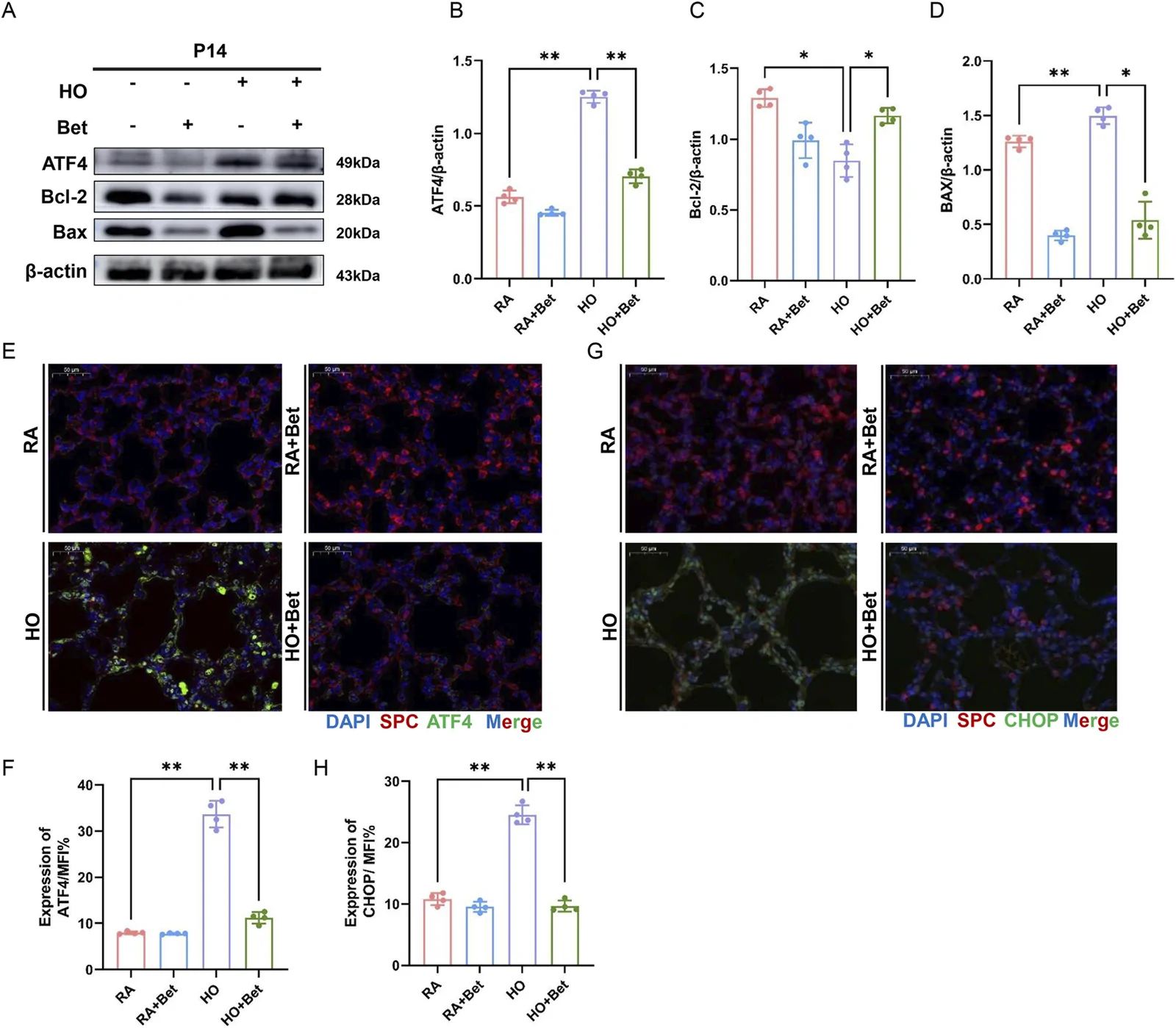
Betaine attenuates integrated stress response and apoptosis of AT2 cells in the lung of mice with BPD. **(A)** Representative Western blot images of ATF4, Bax, and Bcl-2 protein expression in lung tissues from each group at P14. **(B–D)** Quantification of ATF4, Bax, and Bcl-2 protein levels normalized to β-actin by densitometric analysis. Data are presented as mean ± SD; n = 4 per group. **(E)** Representative immunofluorescence images showing ATF4 localization in AT2 cells in lung tissues from each group at P14. Scale bar = 50 μm. **(F)** Quantification of ATF4-positive AT2 cells. Data are presented as mean ± SD; n = 4 per group. **(G)** Representative immunofluorescence images showing CHOP localization in AT2 cells in lung tissues from each group at P14. Scale bar = 50 μm. **(H)** Quantification of CHOP-positive AT2 cells. Data are presented as mean ± SD; n = 4 per group. Data are presented as mean ± SD. *p < 0.05, **p < 0.01 for the indicated comparisons.

### 3.7 Betaine attenuates apoptosis by regulating OPA1-mediated mitochondrial fusion

To investigate the role of OPA1-mediated mitochondrial fusion in the protective effects of betaine, MLE-12 cells were treated with MYLS22 (50 μM), a selective OPA1 inhibitor. Immunofluorescence analysis showed that betaine increased OPA1 expression in MLE-12 cells under hyperoxic conditions, whereas co-treatment with MYLS22 attenuated this effect (Figures 7A,B). Western blot analysis confirmed that betaine increased the protein expression of OPA1 and FAM162A, and this effect was reduced in the presence of MYLS22 (Figures 7C–E). In addition, betaine reduced the Bax/Bcl-2 ratio and cleaved caspase-3, whereas these regulatory effects were weakened by MYLS22 treatment (Figures 7F–H). These findings indicate that betaine attenuates hyperoxia-induced apoptosis in MLE-12 cells by upregulating OPA1-mediated mitochondrial fusion.

**FIGURE 7:**
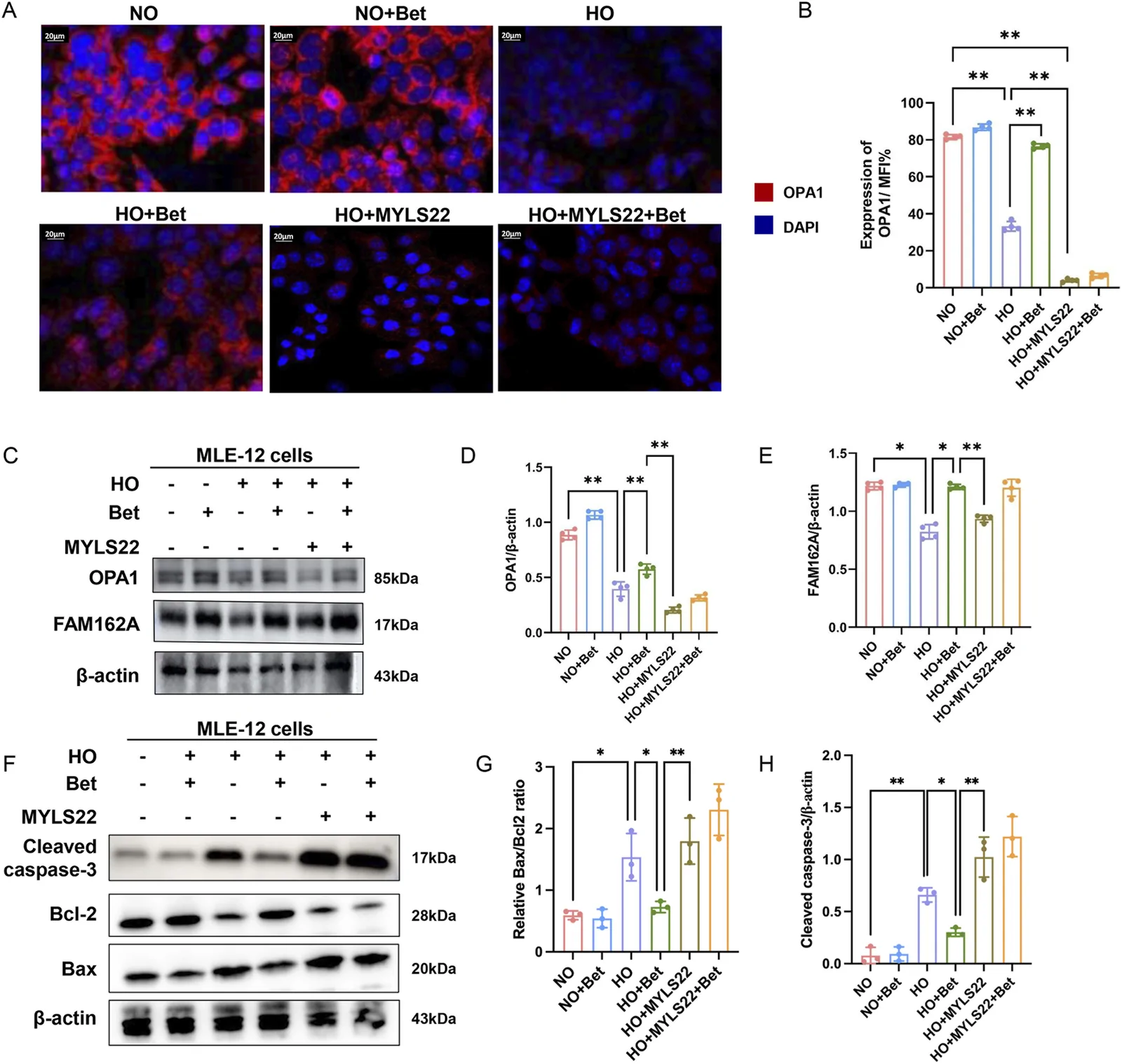
Betaine attenuates apoptosis by regulating OPA1-mediated mitochondrial fusion. MLE-12 cells were randomly divided into six groups: normoxia group (21%O_2_, NO), normoxia with betaine group (21%O_2_, NO + Bet), hyperoxia group (85%O_2_, HO), hyperoxia with betaine group (85%O_2_, HO + Bet), hyperoxia with MYLS22 group (85%O_2_, HO + MYLS22), and hyperoxia with MYLS22 and betaine group (85%O_2_, HO + MYLS22 + Bet). **(A)** Representative immunofluorescence images showing OPA1 expression in MLE-12 cells from each group. Scale bar = 50 μm. **(B)** Quantification of OPA1 expression presented as mean fluorescence intensity (MFI%). Data are presented as mean ± SD; n = 4 per group. **(C)** Representative Western blot images of OPA1 and FAM162 A protein expression in MLE-12 cells from each group. **(D,E)** Quantification of OPA1 and FAM162 A protein levels normalized to β-actin by densitometric analysis. Data are presented as mean ± SD; n = 4 per group. **(F)** Representative Western blot images of Bax and Bcl-2 protein expression in MLE-12 cells from each group. **(G)** Quantification of the Bax/Bcl-2 ratio by densitometric analysis. Data are presented as mean ± SD; n = 3 per group. Data are presented as mean ± SD. *p < 0.05, **p < 0.01 for the indicated comparisons. **(H)** Quantification of cleaved caspase-3 protein levels normalized to β-actin by densitometric analysis. Data are presented as mean ± SD; n = 3 per group.

## 4 Discussion

The present study shows that betaine exhibits a protective effect in hyperoxia-induced lung injury through inhibiting integrated stress response (ISR)-related stress signaling in newborn mice. Mechanistically, betaine preserves mitochondrial homeostasis by restoring OPA1 and FAM162 A expression, thereby alleviating mitochondrial dysfunction, suppressing maladaptive ISR activation, and reducing apoptosis of AT2 cell. These findings identify mitochondrial dynamic imbalance as a critical mechanism underlying hyperoxia-induced alveolar developmental arrest and support the therapeutic potential of betaine in BPD.

Mitochondrial quality control is a coordinated system that maintains mitochondrial integrity through multiple processes, including mitochondrial biogenesis, dynamics, mitophagy, and proteostasis (Liu et al., 2024). Under hyperoxic stress, disruption of mitochondrial homeostasis may lead to the accumulation of dysfunctional mitochondria, impaired bioenergetic function, and excessive oxidative injury. Persistent mitochondrial dysfunction can further activate the ISR, a conserved stress-adaptive pathway that coordinates cellular survival, metabolic remodeling, and fate decisions under stress conditions (Costa-Mattioli and Walter, 2020). Sustained ISR-related signaling, particularly through the ATF4/CHOP axis, may shift cellular responses from adaptation toward apoptosis and pathological tissue injury (Lu et al., 2024). In the present study, hyperoxia reduced mitochondrial content and membrane potential, decreased ATP production, and increased oxidative stress. These changes were accompanied by increased ATF4 and CHOP expression and an altered Bax/Bcl-2 balance in AT2 cells.

Mitochondrial fusion supports the exchange of mitochondrial content and maintains respiratory efficiency, whereas mitochondrial fission facilitates mitochondrial remodeling and the removal of damaged mitochondria (Eisner et al., 2018). Disruption of this balance aggravates mitochondrial depolarization, ATP deficiency, ROS accumulation, and cell death (Giacomello et al., 2020). Dai et al. reported that hyperoxia promoted mitochondrial fission signaling in BPD, whereas inhibition of DRP1 improved pulmonary vascular development and attenuated pulmonary hypertension (Dai et al., 2020). FAM162A, also known as human growth and transformation-dependent protein (HGTD-P), has been implicated in mitochondrial apoptotic cascades, including mitochondrial permeability transition, cytochrome c release, and caspase activation (Lee et al., 2004). In addition, Hsp90-dependent mitochondrial translocation of FAM162 A is required for FAM162 A-induced mitochondrial apoptotic signaling (Kim et al., 2006). Recent evidence has further shown that FAM162 A also participates in mitochondrial structural maintenance, dynamics and bioenergetic regulation, and interacts with OPA1 to regulate long and short OPA1 isoforms (Matamoros et al., 2026). In the present study, FAM162 A was identified as a potential candidate through proteomic analysis and further investigated because of its biological relevance to mitochondrial structural regulation. It should be noted that FAM162 A was not the most statistically significant DEP identified in the proteomic dataset. Among the 56 DEPs, FAM162 A ranked fourth according to fold change but ranked 44th according to nominal *p*-value. Although several DEPs, including Ube2n, Eny2, Syk, Dipk2a, and Tkt, showed stronger statistical significance. FAM162 A was selected as a hypothesis-driven candidate because its localization to the mitochondrial inner membrane and cristae, together with its reported association with OPA1, provided a direct biological connection to mitochondrial fusion regulation and the ultrastructural abnormalities observed after hyperoxia exposure. Other statistically significant DEPs may represent additional molecular pathways involved in hyperoxia-induced lung injury and warrant further investigation. Consistent with this hypothesis, our experimental findings demonstrated that hyperoxia markedly reduced OPA1 and FAM162 A expression both *in vivo* and *in vitro*, suggesting the involvement of impaired mitochondrial fusion-related signaling in hyperoxic lung injury. Consistent with the protein-level changes, hyperoxia significantly reduced FAM162 A mRNA expression, whereas betaine restored FAM162 A mRNA levels under hyperoxic conditions. These findings suggest that hyperoxia-induced downregulation of FAM162 A may occur, at least partially, through transcriptional suppression, and that restoration of FAM162 A expression may contribute to the protective effects of betaine on mitochondrial homeostasis.

Betaine has attracted increasing attention as a metabolic regulator with antioxidant and cytoprotective properties (Zhao et al., 2018). Studies in metabolic disease models have shown that betaine improves methyl metabolism, reduces oxidative stress, and enhances cellular stress tolerance (Rehman and Mehta, 2022; Seo et al., 2024). Growing evidence also indicates that betaine modulates mitochondrial homeostasis. Betaine enhances mitochondrial respiration (Lee, 2015), increases mitochondrial content and oxidative metabolic activity (Zhang et al., 2019), and attenuates mitochondrial dysfunction under toxic stress conditions by preserving ATP production and mitochondrial membrane potential (Im et al., 2013). Recent work further reveals that betaine improves age-related mitochondrial respiratory impairment and preserves skeletal muscle function (Chen et al., 2024). Notably, Kim et al. demonstrated that betaine regulates mitochondrial dynamics by decreasing DRP1 and increasing MFN2, thereby enhancing mitochondrial function and cell survival (Jung Kim, 2018). In pulmonary research, proline betaine, a betaine-related metabolite, promotes angiogenesis and support lung development in BPD (Cili et al., 2025). In this experiment, betaine lowered Bax expression in mice with room air, which suggests that betaine may exert anti apoptotic effect both under normoxic and hyperoxic conditions. However, under high oxygen conditions, the inhibitory effect of betaine on apoptosis becomes more pronounced. Thus, betaine attenuated the apoptosis of AT2 cells in the lung of mice with BPD. Our previous study showed that betaine attenuated hyperoxia-induced lung injury by reducing macrophage pyroptosis (Zhang et al., 2026). Meanwhile, it should be noted that the recovery of mitochondrial membrane potential assessed by JC-1 staining was more modest than the increase in MitoTracker Green fluorescence after betaine treatment under hyperoxic conditions. This difference is biologically plausible because JC-1 reflects mitochondrial membrane potential, whereas MitoTracker Green mainly reflects mitochondrial mass or content. Therefore, betaine may more effectively preserve mitochondrial content than fully restore mitochondrial polarization under the present experimental conditions. These findings suggest a partial, rather than complete, improvement of mitochondrial dysfunction by betaine. In the present study, betaine ameliorated the expression of FAM162 A and OPA1, improved mitochondrial functions, suppressed ISR activation, and ameliorated hyperoxic lung injury in newborn mice.

There are several limitations in this study. First, although our findings suggest that FAM162 A participates in mitochondrial homeostasis, the precise downstream molecular mechanisms remain unclear. Second, the mechanism between OPA1 and ISR activation requires further validation using genetic manipulation approaches. Third, the present study did not directly demonstrate a physical interaction between FAM162 A and OPA1 or establish a direct regulatory effect of FAM162 A on OPA1-mediated mitochondrial fusion. Further studies using co-immunoprecipitation and FAM162 A siRNA/overexpression systems are needed to clarify the direct mechanistic relationship between FAM162 A and OPA1. Finally, whether betaine exerts similar protective effects in preterm infants with BPD requires future investigation.

In conclusion, this study demonstrates that betaine attenuates hyperoxia-induced lung injury by promoting mitochondrial fusion via the FAM162A/OPA1 pathway, suppressing ISR activation, and reducing AT2 cell apoptosis, thereby improving lung development in BPD mice. These findings suggest that betaine may serve as a potential therapeutic agent for BPD.

## 5 Conclusion

This study indicates that betaine improves the mitochondrial fusion of AT2 cells by upregulating the expression of FAM162A/OPA1, reduces integrated stress response-related signaling and the apoptosis of AT2 cells, and improve lung development in BPD mice, providing a new insight for the prevention and treatment of BPD.

## Data Availability

The raw proteomics data has been deposited to the ProteomeXchange Consortium and the code number is PXD084689. All essential data for evaluating the conclusions in the paper are provided in the main text and the Supplementary Information.

## Supplementary material

SUPPLEMENTARY FIGURE S1 Betaine attenuates hyperoxia-induced downregulation of FAM162A mRNA in MLE-12 cells. MLE-12 cells were divided into four groups: normoxia (NO), normoxia + betaine (NO + Bet), hyperoxia (HO), and hyperoxia + betaine (HO + Bet). **(A)** Relative mRNA levels of FAM162A were measured by qPCR and normalized to the normoxia group. Data are presented as mean ± SD; n = 3 per group. **p < 0.01 for the indicated comparisons. Data are presented as mean ± SD; n = 3 per group. **p* < 0.05, ***p* < 0.01 for the indicated comparisons.

SUPPLEMENTARY FIGURE S2 Betaine attenuates hyperoxia-induced apoptosis in MLE-12 cells. MLE-12 cells were divided into four groups: normoxia (NO), normoxia + betaine (NO + Bet), hyperoxia (HO), and hyperoxia + betaine (HO + Bet), hyperoxia + MYLS22 (HO + MYLS22), and hyperoxia + MYLS22 + betaine (HO + MYLS22 + Bet). **(A)** Representative flow cytometry scatter plots showing apoptosis in MLE-12 cells from each group, detected by Annexin V/PI staining. **(B)** Quantification of the apoptosis rate in each group. Data are presented as mean ± SD; n = 6 per group. Data are presented as mean ± SD. **p* < 0.05, ***p* < 0.01 for the indicated comparisons.
