# Supplementary material for: Betaine restores OPA1-mediated mitochondrial fusion in alveolar epithelial type 2 cells and ameliorates hyperoxic lung injury in newborn mice
Source: Front Cell Dev Biol. 2026 Sep 29;14:1937629. doi: 10.3389/fcell.2026.1937629 (PMC13658552; doi:10.3389/fcell.2026.1937629)
Supplement: Supplementary file 1 [file Image1.pdf]

A

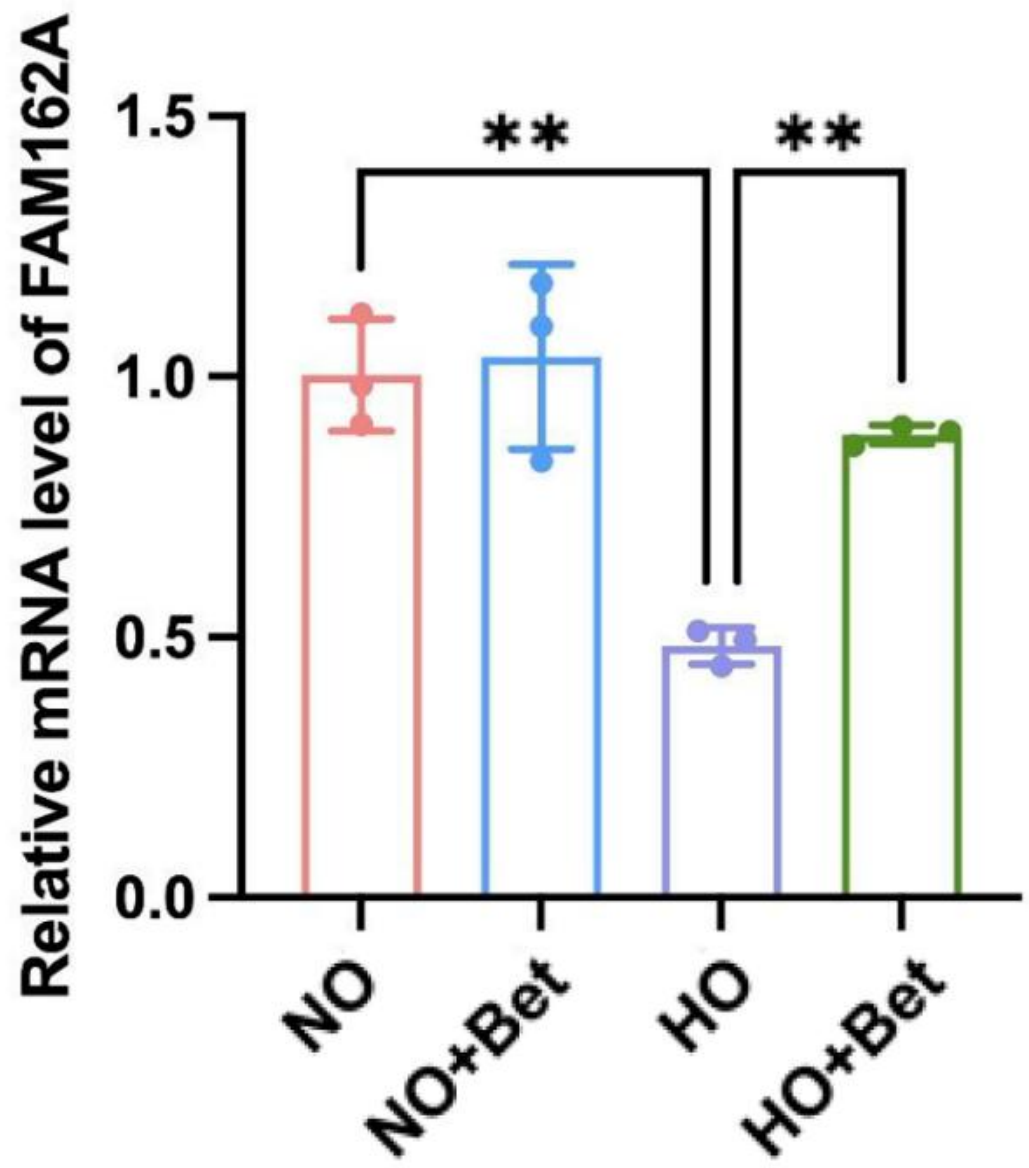

Fig. S1 Betaine attenuates hyperoxia-induced downregulation of FAM162A mRNA in MLE-12 cells

MLE-12 cells were divided into four groups: normoxia (NO), normoxia + betaine (NO + Bet), hyperoxia (HO), and hyperoxia + betaine (HO + Bet).

A. Relative mRNA levels of FAM162A were measured by qPCR and normalized to the normoxia group. Data are presented as mean ± SD; n = 3 per group. **p < 0.01 for the indicated comparisons.

Data are presented as mean ± SD; n = 3 per group. *p < 0.05, **p < 0.01 for the indicated comparisons.

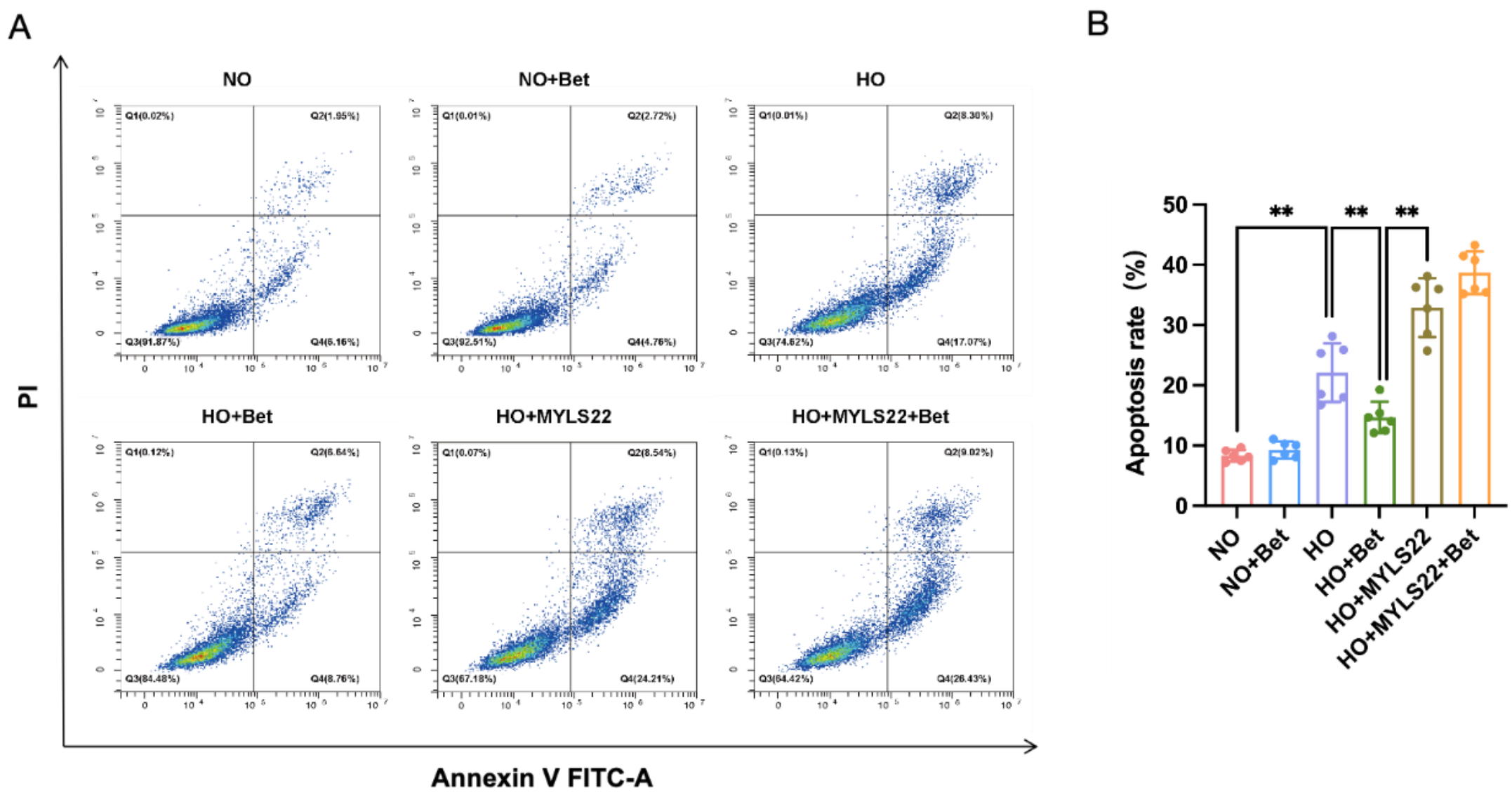

Fig. S2 Betaine attenuates hyperoxia-induced apoptosis in MLE-12 cells.

MLE-12 cells were divided into four groups: normoxia (NO), normoxia + betaine (NO + Bet), hyperoxia (HO), and hyperoxia + betaine (HO + Bet), hyperoxia + MYLS22 (HO + MYLS22), and hyperoxia + MYLS22 + betaine (HO + MYLS22 + Bet).

A. Representative flow cytometry scatter plots showing apoptosis in MLE-12 cells from each group, detected by Annexin V/PI staining.

B. Quantification of the apoptosis rate in each group. Data are presented as mean ± SD; n = 6 per group.

Data are presented as mean ± SD. $*p < 0.05$, $**p < 0.01$ for the indicated comparisons.
