# Supplementary material for: Betaine restores OPA1-mediated mitochondrial fusion in alveolar epithelial type 2 cells and ameliorates hyperoxic lung injury in newborn mice
Source: Front Cell Dev Biol. 2026 Sep 29;14:1937629. doi: 10.3389/fcell.2026.1937629 (PMC13658552; doi:10.3389/fcell.2026.1937629)
Supplement: Supplementary file 2 [file Image2.pdf]

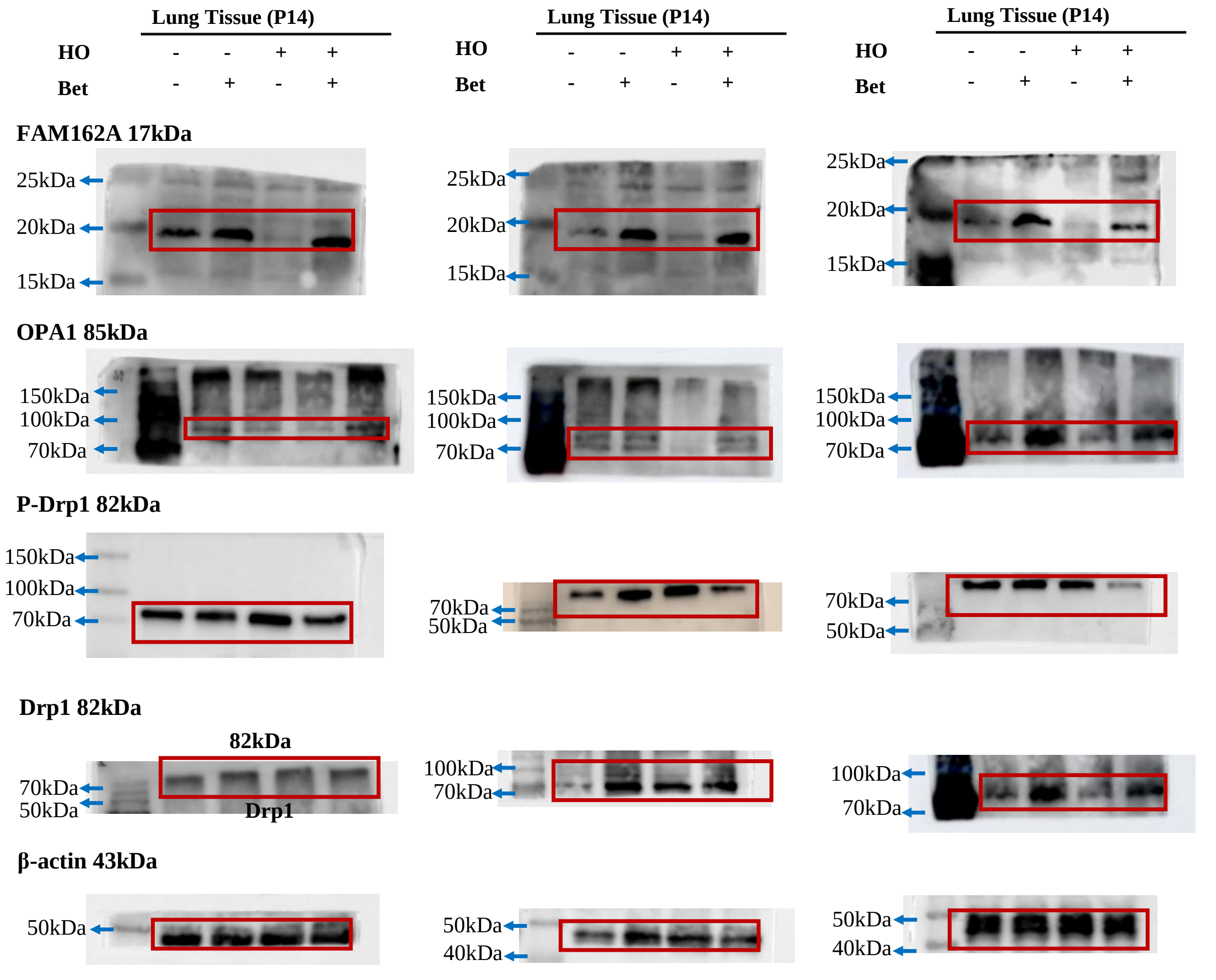

Supplemental Figure. Uncropped immunoblots of the different figures 5A and 5C. Immunoblots related to Figure 5A and 5C include FAM162A, OPA1, P-Drp1, Drp1 and β-actin from lung tissue lysates of mice.

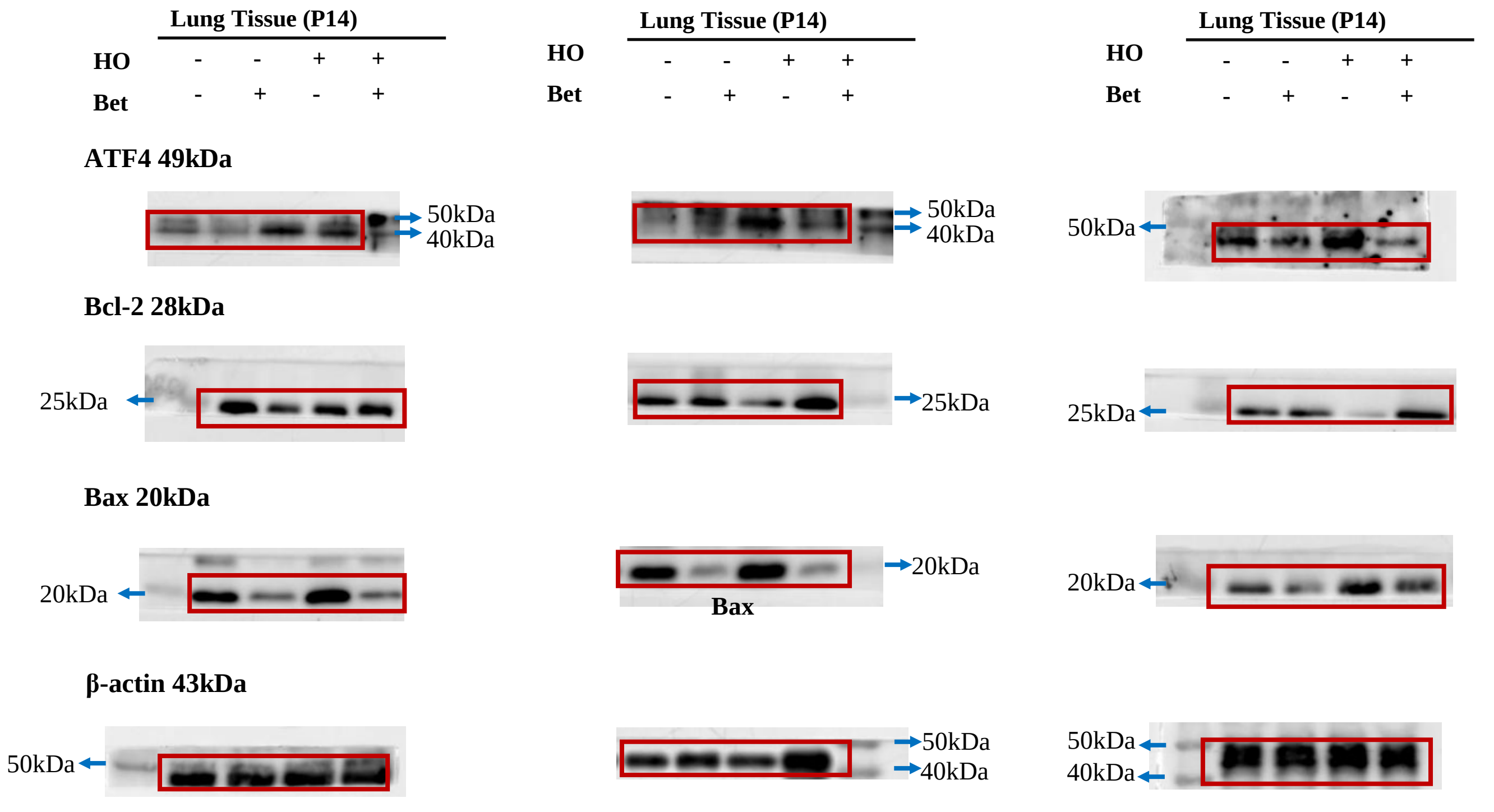

Supplemental Figure. Uncropped immunoblots of the different figures 6A. Immunoblots related to Figure 6A include ATF4, Bcl-2, Bax and β-actin from lung tissue lysates of mice.

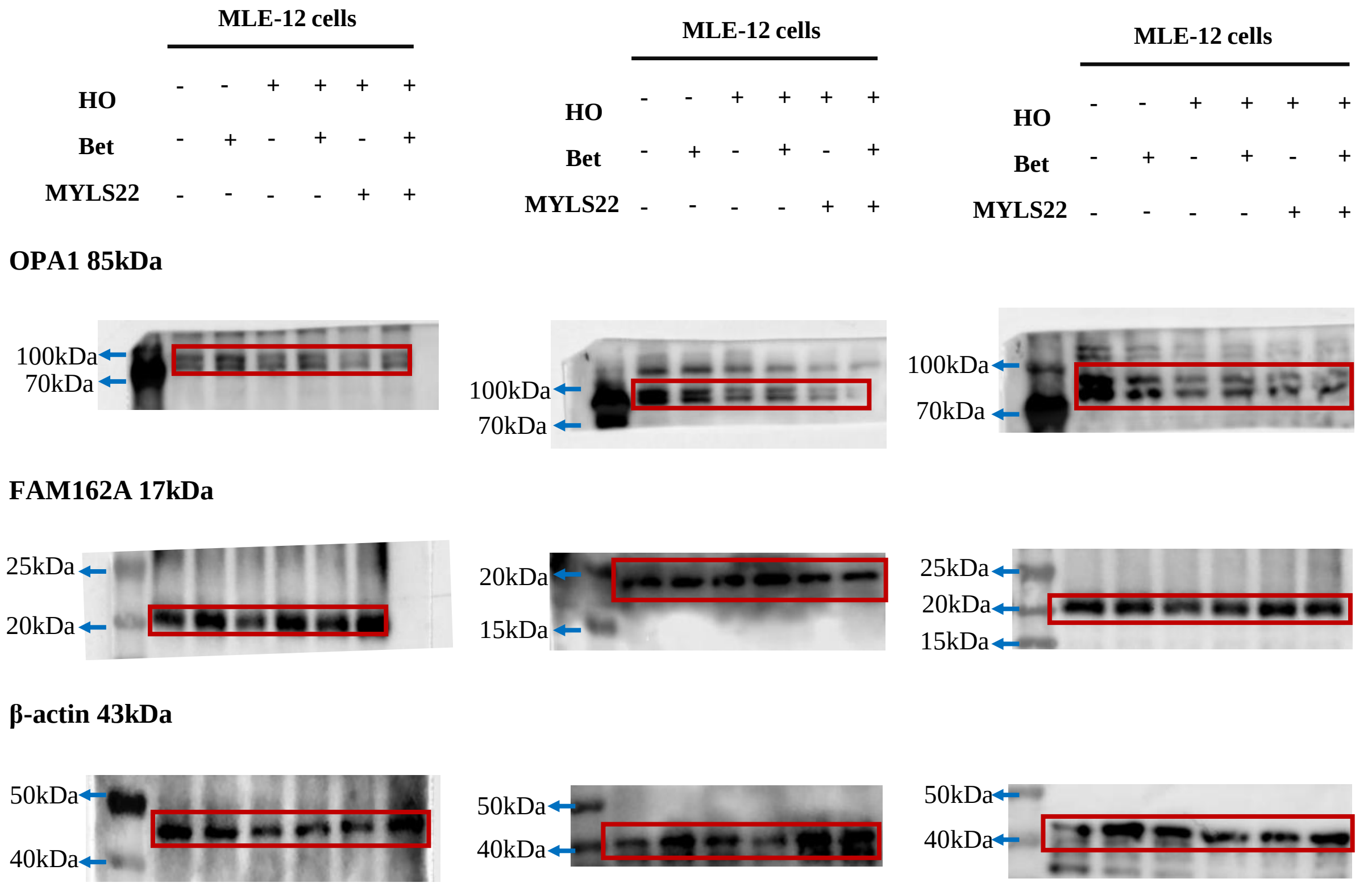

Supplemental Figure. Uncropped immunoblots of the different figures 7C. Immunoblots related to Figure 7C include FAM162A, OPA1 and β-actin from MLE-12 cells.

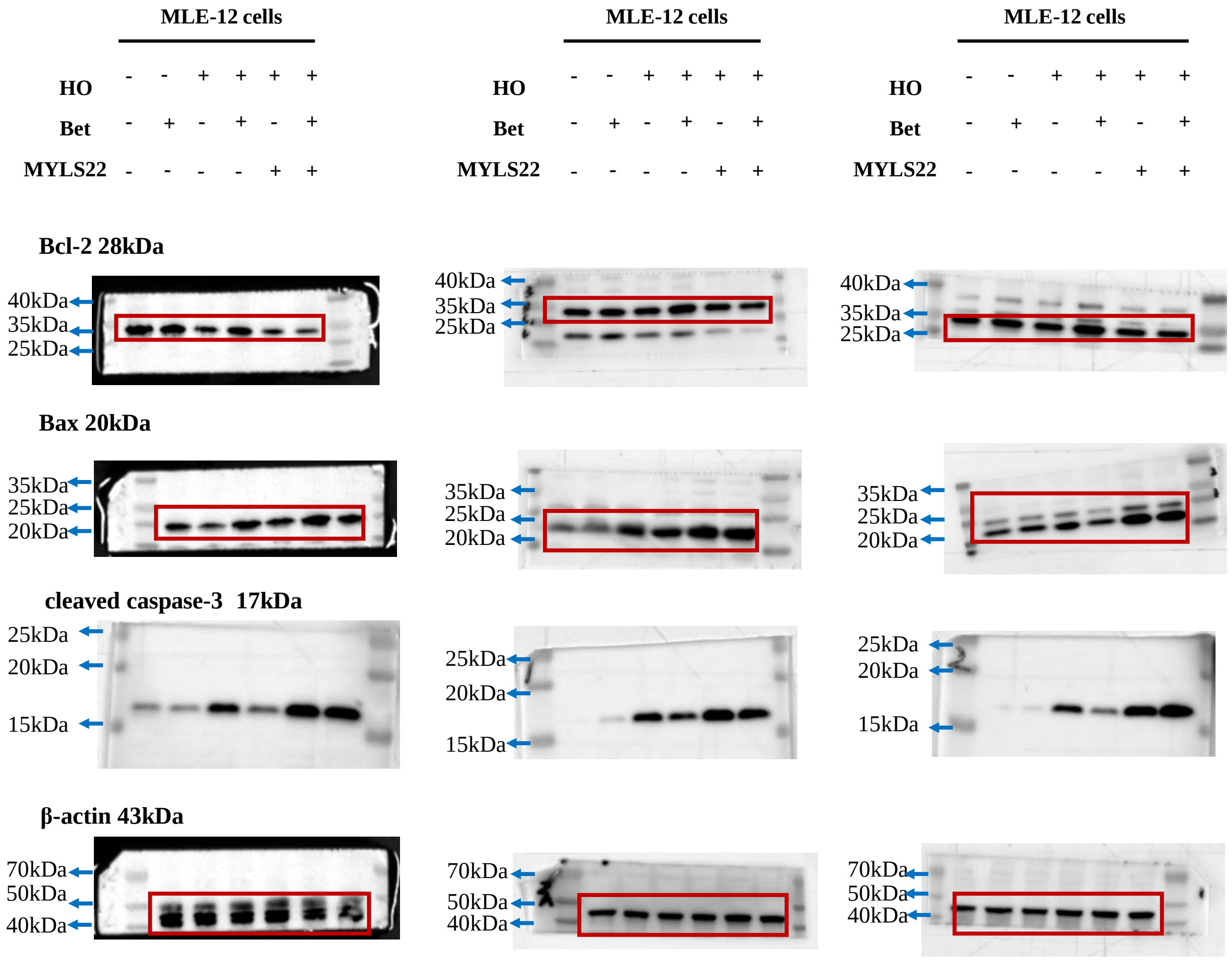

Supplemental Figure. Uncropped immunoblots of the different figures 7F. Immunoblots related to Figure 7F include Bcl-2, Bax, cleaved caspase-3 and β-actin from MLE-12 cells.
